# The CX3CL1-CX3CR1 chemokine axis can contribute to tumor immune evasion and blockade with a novel CX3CR1 monoclonal antibody enhances response to anti-PD-1 immunotherapy

**DOI:** 10.3389/fimmu.2023.1237715

**Published:** 2023-09-13

**Authors:** Apoorvi Chaudhri, Xia Bu, Yunfei Wang, Michael Gomez, James A. Torchia, Ping Hua, Shao-Hsi Hung, Michael A. Davies, Gregory A. Lizee, Ulrich von Andrian, Patrick Hwu, Gordon J. Freeman

**Affiliations:** ^1^ Department of Medical Oncology, Dana-Farber Cancer Institute, Boston, MA, United States; ^2^ Department of Melanoma Medical Oncology, Division of Cancer Medicine, The University of Texas MD Anderson Cancer Center, Houston, TX, United States; ^3^ The University of Texas MD Anderson Cancer Center UTHealth Graduate School of Biomedical Sciences, Houston, TX, United States; ^4^ Department of Medicine, Harvard Medical School, Boston, MA, United States; ^5^ Department of Clinical Science, H. Lee Moffitt Cancer Center & Research Institute, Tampa, FL, United States; ^6^ Department of Immunology & HMS Center for Immune Imaging, Harvard Medical School, Boston, MA, United States; ^7^ Ragon Institute of MGH, MIT and Harvard, Cambridge, MA, United States

**Keywords:** CX3CR1, CX3CL1, PD-1, tumor immune evasion, cancer immunotherapy

## Abstract

CX3CL1 secreted in the tumor microenvironment serves as a chemoattractant playing a critical role in metastasis of CX3CR1 expressing cancer cells. CX3CR1 can be expressed in both cancer and immune-inhibitory myeloid cells to facilitate their migration. We generated a novel monoclonal antibody against mouse CX3CR1 that binds to CX3CR1 and blocks the CX3CL1-CX3CR1 interaction. We next explored the immune evasion strategies implemented by the CX3CL1-CX3CR1 axis and find that it initiates a resistance program in cancer cells that results in 1) facilitation of tumor cell migration, 2) secretion of soluble mediators to generate a pro-metastatic niche, 3) secretion of soluble mediators to attract myeloid populations, and 4) generation of tumor-inflammasome. The CX3CR1 monoclonal antibody reduces migration of tumor cells and decreases secretion of immune suppressive soluble mediators by tumor cells. In combination with anti-PD-1 immunotherapy, this CX3CR1 monoclonal antibody enhances survival in an immunocompetent mouse colon carcinoma model through a decrease in tumor-promoting myeloid populations. Thus, this axis is involved in the mechanisms of resistance to anti-PD-1 immunotherapy and the combination therapy can overcome a portion of the resistance mechanisms to anti-PD-1.

## 1 Introduction

Immunotherapy with anti-PD-1, PD-L1 and CTLA-4 antibodies has revolutionized cancer treatment (1, 2). Anti-PD-1 immunotherapy is FDA approved in multiple cancer types; however, resistance mechanisms result in only a moderate percentage of clinical responses (3). The abundance of immune suppressive myeloid cells is a major resistance mechanism to anti-PD-1 in multiple tumor types (4, 5). Aberrant myelopoiesis is a hallmark event in cancer where myeloid cells with immune suppressive properties infiltrate the tumor microenvironment (6). Thus, combination therapies that block the generation and maintenance of immune suppressive myeloid populations are promising approaches to enhance clinical responses to anti-PD-1 therapy (5, 7, 8).

Non-responders to anti-PD-1 in non-small cell lung cancer showed an increase in the plasma concentrations of the CX3CR1 ligand, CX3CL1 (9). An immune suppressive myeloid population defined as CX3CR1+CD206+ may strongly influence the outcome of the response to anti-PD-1 therapy since tumor-CX3CR1+CD206+ myeloid cells were reduced after response to anti-PD-1 in a T3 sarcoma mouse model (10). In summary, the failure to reduce CX3CR1+ myeloid populations may result in non-response and resistance to anti-PD-1 immunotherapy.

CX3CR1 binds to its ligand, CX3CL1 (also known as fractalkine or neurotactin), which has membrane-bound and shed forms. The CX3CL1-CX3CR1 axis promotes chemotaxis of CX3CR1+ cells towards soluble CX3CL1 as well as adhesion of CX3CR1+ cells to membrane-bound CX3CL1 (11, 12). CX3CR1 activation induces signaling events (13).

Following interaction with CX3CL1, CX3CR1 signaling supports tumorigenesis through several mechanisms: 1. CX3CL1 in the tumor milieu promotes an influx of CX3CR1+ myeloid cells; a hallmark event of aberrant myelopoiesis, 2. CX3CL1 in the tumor milieu promotes migration of multiple CX3CR1+ tumor types such as breast cancer, prostate cancer, CLL, neuroblastoma, glioblastoma, pancreatic ductal carcinoma (PDAC), colon carcinoma, gastric cancer, skin cancer, lung cancer, osteosarcoma, melanoma, multiple myeloma, and bladder cancer (14–18), and 3. CX3CL1 in the tumor milieu promotes activation of several oncogenic pathways following interaction with CX3CR1 in tumors (19–36).

CRISPR deletion of CX3CR1 or its blockade in human tumor lines of PDAC, breast cancer, prostate cancer, bladder cancer and glioblastoma results in a decrease in the ability of tumor cells to migrate and metastasize (34, 37). CX3CR1 deficient mice show a reduction in tumor infiltrating macrophages in SL4 colon carcinoma and skin cancer model (15, 38). A small molecule antagonist to CX3CR1 has shown efficacy in preclinical models of breast cancer (34); however, a challenge of small molecule CX3CR1 antagonists is that they can target several related GPCRs (G Protein-coupled receptors) and are not unique to CX3CR1. An antibody approach might have advantages due to its specificity for CX3CR1, and better receptor engagement based on avidity wherein a higher receptor expression will be required for effective cell depletion by ADCC (39). Together, this led us to investigate whether blockade of the CX3CL1-CX3CR1 axis using an antibody approach would augment the response to anti-PD-1 therapy and increase the number of responders in preclinical models whilst offering clinical translatability.

We generated a novel monoclonal antibody that binds to CX3CR1 with high affinity, blocks its interaction with CX3CL1 and antagonizes the immune-suppressive signals of this axis. We observe an improvement in response to anti-PD-1 therapy in an immunocompetent syngeneic mouse model of colon carcinoma. Mechanistically, combined PD-1 and CX3CR1 blockade reduces the migration of tumor cells, decreases the abundance of immune suppressive myeloid cells in the tumor, increases mature macrophages in the tumor and reduces secretion of soluble mediators from the tumor. The CX3CR1 monoclonal antibody can target both tumor and myeloid cells; inhibit tumor signals that recruit myeloid populations and inhibit immune-suppressive pathways regulated through this axis. In summary, the CX3CR1 antibody can mitigate CX3CL1-CX3CR1 mediated pro-tumorigenic effects of aberrant myelopoiesis during cancer progression.

## 2 Methods

### 2.1 CX3CR1 monoclonal antibody

The monoclonal antibodies for mouse CX3CR1, clones 455.1C11, and 455.8H12 were made by immunizing CX3CR1 knockout mice of C57BL/6 background with mouse CX3CR1 cDNA expression plasmid DNA as described in Latchman et al., 2001 (40) (RRID: IMSR_JAX:005582) and 293T cells transiently transfected with mouse CX3CR1 cDNA. The CX3CR1 mutant mice were originally provided by Dr. Dan Littman (New York University) (41). Hybridomas were screened for reactivity with Jurkat and 300 cells transfected to express mouse CX3CR1 and a lack of reactivity with un-transfected Jurkat cells and 300.19 cells. These monoclonal antibodies are of IgG2c isotype, an isotype found in C57BL/6 mice, the strain of origin of the CX3CR1 knockout. The binding affinities of these CX3CR1 mAbs were tested with 300.19 cells transfected to express mouse CX3CR1. The detection reagent was Goat anti-mouse IgG-PE Cat #1036-09 1036-09 (10 µg/ml). The isotype control was mIgG2c Cat #0122-01.

### 2.2 Cells and cell culture

CT26 colon carcinoma was purchased from ATCC (ATCC Cat# CRL-2638, RRID : CVCL_7256). CT26 were maintained in RPMI-1640 with 10% heat inactivated FCS, 1% glutamax, 1% pen-strep in 5% CO_2_. Jurkat cells transfected with mouse CX3CR1 were made in our laboratory and maintained in RPMI-1640 media with 5 µg/ml puromycin. 300 cells transfected with mouse CX3CR1, and 300 cells transfected with mouse PD-L1 were made in our laboratory and maintained in RPMI-1640 media with 5 µg/ml puromycin and 50 µM 2-mercaptoethanol in 5% CO_2._ CHO cells transfected to express human CX3CR1 were purchased from Sigma Aldrich Cat #HTS015RTA.

All cell lines used were tested and negative for mycoplasma.

### 2.3 Recombinant protein and antibody for *in vitro* assays

The isotype controls MOPC21 (mIgG1), and C1.18.4 (mIgG2a) were purchased from BioXcell. Isotype mIgG2c, Goat anti-mouse IgG-PE, goat anti-human IgG-PE (Southern Biotech Cat# 2040-09, RRID : AB_2795648), and goat anti-human IgG-Alexa647 (multi species adsorbed) (Southern Biotech Cat# 2040-31, RRID : AB_2795651) were purchased from Southern Biotech. CX3CL1-human IgG1 Fc fusion protein for blocking assays was a kind gift from Dr. Ulrich von Andrian, Harvard Medical School. CX3CL1 recombinant protein was purchased from Novus Biologicals (cat NBP2-35038).

### 2.4 Blocking assays with CX3CR1 mAbs

The capacity of anti-CX3CR1 mAbs to block binding of CX3CL1 to CX3CR1 was tested by incubating Jurkat-mCX3CR1 cells with anti-CX3CR1 mAb hybridoma supernatant for 30 min, followed by addition of CX3CL1-hFc (at 2 µg/ml). The binding of CX3CL1-Fc was detected using Goat anti human IgG-PE (mouse adsorbed) (Southern Biotech Cat# 2043-09, RRID : AB_2795669) at 5 µg/ml. Isotype control was mIgG1 (BioXcell Cat# BE0083 RRID:1107784). Acquisition was performed on BD FACS LSR Fortessa, and data was analyzed using Flow Jo version 10 (RRID : SCR_008520). Antibody concentrations of anti-mouse CX3CR1 antibodies in hybridoma supernatants were determined by indirect ELISA. ELISA plates (Costar #3369) were coated with 2 µg/ml unlabeled goat anti-mouse Ig(H+L) antibody overnight. The next day, plates were washed, blocked with 1% BSA in PBS for one hour and then washed. Hybridoma supernatants and dilutions were added to individual wells, incubated at 37°C for 1 hour and then washed three times. Goat anti-mouse IgG2c HRP-conjugated secondary antibody (Southern Biotech Cat# 1078-05, RRID : AB_2794462) was added to the wells, incubated at 37°C for 1 hour and then washed three times. TMB substrate was added to develop the color and plates were analyzed using a SpectraMax 190 microplate reader. Purified mouse IgG2c was used to construct a standard curve to calculate the antibody concentrations in supernatants.

The capability of anti-CX3CR1 mAbs clones 1C11 and 8H12 to block binding of each other to CX3CR1 was tested by incubating Jurkat-mCX3CR1 cells with anti-CX3CR1 mAb clone 1C11 for 30 min, followed by addition of anti-CX3CR1 mAb clone 8H12-PE at 1 µg/ml. Isotype control was C1.18 mIgG2a. Acquisition was performed on BD FACS LSR Fortessa and data was analyzed using Flow Jo version 10 (RRID : SCR_008520).

### 2.5 Antagonistic and agonistic assays

Agonistic and antagonistic assays for CX3CL1-CX3CR1 axis activity were performed using the PathHunter eXpress mCX3CR1 CHO-K1 β-Arrestin GPCR Assay kit (Eurofin Discovery X; catalog 93-0702E2MCP2M) following the manufacturer’s protocol. In the antagonistic assay, mouse CX3CR1 CHO-K1 reporter cells were incubated with anti-CX3CR1 mAb clone 1C11 for 30 minutes at 37°C followed by addition of agonist CX3CL1 (Novus Biologicals; cat NBP2-35038) at 500 ng/ml for 90 minutes at 37°C. Isotype control was mIgG2c. Detection reagent (assay buffer and substrate) was then added, and luminescence was measured on Spectra Max M3 instrument. In the agonistic assay, mouse CX3CR1 CHO-K1 reporter cells were incubated with agonist CX3CL1 (Novus Biologicals; cat NBP2-35038) or with anti-CX3CR1 mAb clone 1C11 for 90 minutes at 37°C. Detection reagent (assay buffer and substrate) was then added followed by luminescence measurement.

### 2.6 CX3CL1-CX3CR1 migration assays

Tumor cell migration assays were performed using Abcam cell migration/chemotaxis assay utilizing a Boyden chamber with an 8 µm membrane (Abcam; catalog ab235673) as per the manufacturer’s protocol. Briefly, CX3CR1 expressing CT26 tumor cells were cultured in serum free media for 24 hours. Tumor cells were incubated with CX3CR1 mAb (clone 1C11) at 10 µg/ml for 30 minutes at 37°C. The chemoattractant CX3CL1 (Novus Biologicals; catalog NBP2-35038) (at 100 ng/ml) was added to the bottom chamber and mAb-treated or control tumor cells were added to the top of the Boyden chamber. The chamber was incubated for 24 hours at 37°C followed by addition of cell dissociation solution and fluorescence measurement using Spectra max M3 instrument.

### 2.7 Tumor cell expression of CX3CR1 and PD-L1

The CT26 tumor cell line was assayed with anti-mouse antibodies; CX3CR1 antibody (clone 1C11) made in our laboratory, CX3CL1 antibody (R and D Systems Cat# MAB571, RRID : AB_2087125), PD-L1 antibody (BioLegend Cat# 329737, RRID : AB_2617009). The corresponding isotypes were used as controls.

### 2.8 Tumor cell expression of CX3CR1 on treatment with pro-inflammatory cytokines IFN-γ and TNF-α

CT26 tumor cell line was treated with mouse IFN-γ (Peprotech; catalog 315-05) and TNF-α (R&D systems; catalog 410-MT-010) at 100 ng/ml for 24 hours. Cells were subsequently assayed for expression of CX3CR1 by flow cytometry.

### 2.9 TCGA analysis and CIBERSORT analysis

The gene expression values of TCGA and GTEx samples were obtained from UCSC Toil RNA-Seq TOIL Recompute Compendium (42). The TCGA clinical information was obtained from GDC Data Portal. The GTEx healthy tissues were matched to TCGA cancer types for comparison (43). The TPM values were log2-transformed with a pseudo count of 1 for visualization of gene expression profiles. The CIBERSORT immune fractions were obtained from the Immune Landscape of Cancer analysis (44). The cohort of each cancer type was split into CX3CR1 high and low groups based on the median value. R package *limma* was used to infer the significance between the fractions (45). R package *survival* was used for the survival analysis.

### 2.10 Mouse *in vivo* tumor immunotherapy experiments

BALB/cJ mice aged 6-8 weeks were purchased from Jackson Laboratory (RRID : IMSR_JAX:000651) and housed in a barrier facility. CT26 tumor cells were subcutaneously injected at 0.2 million/mouse in the right flank of BALB/cJ mice. The antibody treatments with anti-mouse PD-1 (clone 29F.1A12) (Bio X Cell Cat# BE0273, RRID : AB_2687796), anti-mouse CX3CR1 (clone 1C11), rat IgG2a (clone 2A3) (Bio X Cell Cat# BE0089, RRID : AB_1107769) were started in randomized mice on day 7 post tumor cell injection for a series of 5 treatments once every 3 days with 200 µg antibody per mouse injected intraperitoneally per treatment. Mice were monitored for tumor growth using digital calipers, with tumor size calculated as volume (L X W^2^ X 0.5) in mm^3^. Mice were euthanized based on the end point criteria of a tumor diameter more than 20 mm or ulceration in the tumor with ulceration diameter of more than 10 mm. For memory re-challenge experiments, 0.2 million CT26 tumor cells were subcutaneously injected in the opposite (left) flank, and mice were monitored for tumor growth and survival. Here, mice aged 6-8 weeks with no prior antibody treatment were included as a control. Animal experiments were performed as per the IACUC guidelines of the Dana-Farber Cancer Institute.

### 2.11 Tumor infiltrating lymphocyte isolation

For study of tumor immune infiltrates in the untreated cohort, tumors were harvested when the tumor diameter reached a size greater than 0.5-1 cm. For the study of antibody mediated mechanistic effects, tumors were harvested on day 12 after two antibody treatments. Tumors were pooled with two-three tumors to make one sample and increase the per sample cell yield as required for data acquisition. Once harvested, tumors were minced, followed by suspension in a disaggregation buffer (1.5 mg/ml collagenase IV (Sigma Aldrich; cat C5138), 200 U/mL DNase (Roche; cat 04536282001) in HBSS without Ca^++^/Mg^++^) and incubated at 37°C for 30 minutes with agitation. Cells were then subjected to mechanical disruption and filtered to obtain a single cell suspension. Red blood cell lysis was performed using ACK lysis buffer (Gibco) when needed. Spleens were isolated from tumor bearing mice. Splenocytes were harvested from spleen using gentle disruption of the tissue. Red blood cell lysis was performed on the cell suspension using ACK lysis buffer (Gibco) and the cells were filtered to obtain single cells. Cells were Fc blocked by incubating with 10 µg/ml of anti-mouse CD16/32 antibody (BioLegend Cat# 101302, RRID : AB_312801) for 15 minutes and stained with fluorochrome-conjugated antibodies against cell surface markers for 30 min. After cell surface staining, the cells were fixed and permeabilized using FOXP3 transcription factor fixation and permeabilization kit (eBioscience; catalog 00-5521-00) to determine the expression of intracellular markers. The fluorochrome antibodies used are detailed in **Supplementary Table 1**. Acquisition was performed on BD FACSymphony and BD LSR Fortessa. Samples that failed to acquire sufficient cell numbers could not be used. Data analysis was performed using Flow Jo version 10 (RRID : SCR_008520).

### 2.12 RNA sequencing

Tumor cells were treated with CX3CL1 (Novus Biologicals; cat NBP2-35038) at 2 µg/ml for 8 hours and RNA was prepared using RNAeasy kit (Qiagen Catalog 74104). Following incubation, total RNA was prepared using RNAeasy kit (Qiagen Catalog 74104).

RNA sequencing was performed at the Molecular Biology Core Facility, Dana-Farber Cancer Institute. Libraries were prepared using Roche Kapa mRNA HyperPrep strand specific sample preparation kits from 200ng of purified total RNA (tumor sample) or Takara SmartSeq v4 reagents from 1ng of RNA (MDSC sample) according to the manufacturer’s protocol on a Beckman Coulter Biomek i7. The finished dsDNA libraries were quantified by Qubit fluorometer and Agilent TapeStation 4200. Uniquely dual indexed libraries were pooled in an equimolar ratio and shallowly sequenced on an Illumina MiSeq to further evaluate library quality and pool balance. The final pool was sequenced on an Illumina NovaSeq 6000 targeting 40 million 150bp read pairs per library at the Dana-Farber Cancer Institute Molecular Biology Core Facilities.

### 2.13 RNA sequencing analysis

Fastq files were generated using Illumina bcl2fastq v2.20 software. Sequenced reads were aligned to the UCSC mm10 reference genome assembly and gene counts were quantified using STAR (v2.7.3a) (46). Differential gene expression testing was performed by DESeq2 (v1.22.1) (47). RNAseq analysis was performed using the VIPER Snakemake pipeline (48).

### 2.14 Cytokine and chemokine multiplex analysis

Tumor cells were treated with or without CX3CR1 antibody (clone 1C11) at 10 µg/ml for 30 minutes at 37°C followed by CX3CL1 (Novus Biologicals; cat NBP2-35038) at 2 µg/ml for 24 hours. Supernatants were collected and analyzed for quantification of chemokines and cytokines using Discovery assays (Millipore, USA) and were performed by Eve Technologies (Alberta, Canada) as per the manufacturer’s instructions. The data was acquired on the Luminex 200 system.

### 2.15 Real-time PCR

Tumor cell lines were treated with mouse IFN-γ (Peprotech, cat 315-05) and TNF-α (R&D systems; catalog 410-MT-010) at 100 ng/ml for 8 hours. Tumor cells were treated with or without CX3CR1 antibody (10 µg/ml) for 30 minutes at 37°C followed by CX3CL1 (Novus Biologicals; cat NBP2-35038) at 2 µg/ml for 8 hours. RNA was isolated using RNeasy kit (Qiagen catalog 74104) using manufacturer’s protocol and cDNA was made using Bio-Rad cDNA kits. mRNA was quantified using Taqman qPCR (Applied Biosystems, catalog 4304437). The assay was run on Applied Biosystems Quant Studio 6 real time PCR system. Expression levels were normalized to GAPDH. Primers were purchased from Applied Biosystems; mouse PD-L1 (Mm03048248_m1), mouse IL-6 (Mm00446190_m1), mouse NLRP3 (Mm00840904_m1) and mouse GAPDH (Mm99999915_g1).

### 2.16 Statistics

Statistical analysis was performed using Graph-pad Prism version 9 software (RRID : SCR_002798). Data was presented as mean averages with standard error of the mean (SEM). The statistical analysis between two groups was performed using the t test. Statistical analysis between more than two groups was performed using one-way ANOVA with multiple comparisons performed using Tukey’s test. Statistical analysis in the Kaplan Meier survival curves was performed using log-rank (Mantel-Cox) test. P values less than 0.05 were significant and were defined as *p<0.05, **p<0.01, ****p<0.001, ****p<0.0001.

## 3 Results

### 3.1 Characterization of anti-mouse CX3CR1 monoclonal antibody

**Figure 1:**
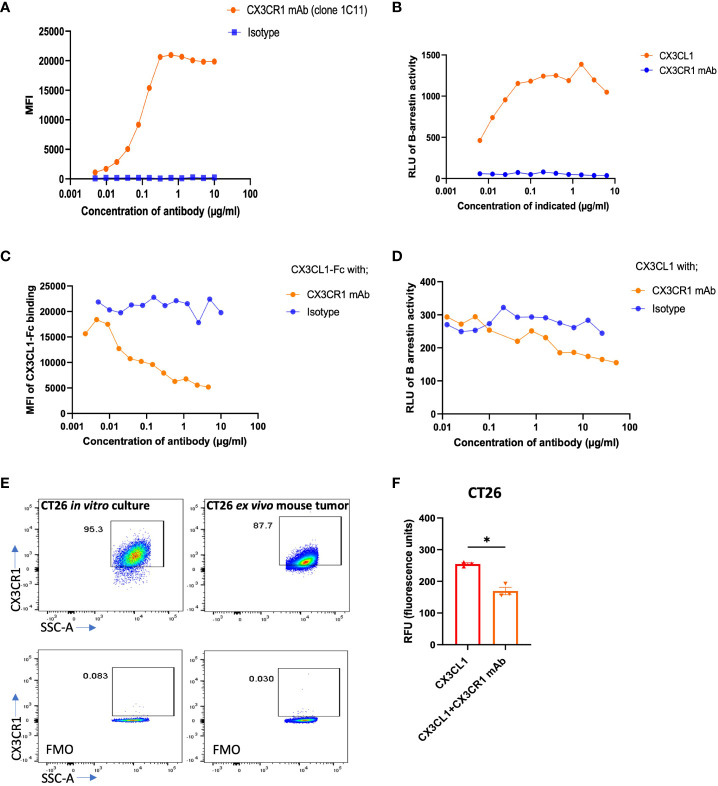
Binding, blocking, and signaling of CX3CR1 mAb. **(A)** FACS analysis of CX3CR1 mAb binding to 300 cells stably expressing mouse CX3CR1. **(B)** Agonist activity of CX3CL1 recombinant protein and of CX3CR1 mAb on CX3CR1-reporter CHO cells. **(C)** CX3CR1 mAb hybridoma supernatant blocking of CX3CL1-hFc (2 µg/ml) fusion protein binding to Jurkat cells stably expressing mouse CX3CR1. **(D)** Antagonistic activity of CX3CR1 mAb on CX3CL1 (500 ng/ml) signaling in CX3CR1-reporter CHO cells. **(E)** FACS analysis of CX3CR1 expression on CT26 from *in vitro* culture and from excised mouse tumor tissue. **(F)** CX3CR1 mAb (10 µg/ml) blockade of CT26 migration towards CX3CL1 (100 ng/ml) (n=3). Data representative of two independent experiments. Data as standard error of the mean, paired t-test, *p<0.05.

We generated monoclonal antibodies (mAb) that recognize mouse (m) CX3CR1 by immunizing CX3CR1 knockout mice and screening for reactivity with mCX3CR1 transfected Jurkat and 300.19 cells and a lack of reactivity with un-transfected cells. We used mCX3CR1 transfected cells as a tool to determine the binding affinity and biological activity of our CX3CR1 mAb. The binding assay showed that the CX3CR1 mAb 455.1C11 recognized mCX3CR1 in a concentration dependent manner with an EC-50 value of 0.09 µg/ml indicating an apparent binding affinity of 0.6 nM while mIgG1 isotype control (MOPC21) did not show binding to 300 mCX3CR1 cells (**Figure 1A**). The 0.6 nM binding affinity of the CX3CR1 mAb is higher than the reported affinity of 0.91 nM for human CX3CL1 ligand binding to CX3CR1 receptor (49).

### 3.2 Antagonistic and agonist activities of CX3CR1 monoclonal antibody

We tested whether the CX3CR1 antibody had agonist or antagonistic activity when bound to CX3CR1 on the cell surface using CHO cells transfected to express mouse CX3CR1 and a β-gal reporter. In these cells, CX3CR1 is coupled to the enzyme donor component of β-gal enzyme and β-arrestin is coupled to the enzyme acceptor component of β-gal enzyme (Eurofin Discovery X). Recruitment of β-arrestin to CX3CR1 generates active β-gal enzyme that emits luminescence as a surrogate of CX3CR1 activation. To confirm the fidelity of the assay, we first showed that CX3CL1 led to β-arrestin recruitment and induced a β-gal signal in a concentration dependent fashion (**Figure 1B**). When CHO-mCX3CR1 cells were treated with the CX3CR1 monoclonal antibody in the absence of CX3CL1, we did not observe a luminescence signal, indicating an absence of β-arrestin recruitment to CX3CR1 (**Figure 1B**) and that the CX3CR1 mAb did not agonize the receptor.

We next performed a blocking assay to determine if our CX3CR1 mAb (1C11) had the ability to block the binding of CX3CL1 to CX3CR1. Jurkat-mCX3CR1 cells were incubated with the CX3CR1 mAb followed by addition of CX3CL1-Fc fusion protein. The 1C11 mAb blocked the CX3CL1-CX3CR1 ligand-receptor interaction in a concentration dependent fashion (**Figure 1C**). As expected, the isotype control was unable to block this interaction. We pre-treated CHO-mCX3CR1-β-gal reporter cells with the CX3CR1 mAb, followed by 500 ng/ml CX3CL1, and observed an antibody concentration dependent blockade of CX3CR1 activation and β-arrestin recruitment (**Figure 1D**) which was not seen when the cells were treated with isotype control. Thus, the CX3CR1 mAb can inhibit the functional activity of CX3CR1. Through *in silico* analysis based on the known co-crystal structure of hCX3CL1 in complex with hCX3CR1, we predicted interactions between mCX3CL1 and mCX3CR1 and between the 1C11 mAb and mCX3CR1. This analysis identified 10 residues in mCX3CR1 that make contact with both mCX3CL1 and the 1C11 mAb (**Supplementary Material**). These findings are consistent with the experimentally determined blockade of mCX3CL1 binding to mCX3CR1 by the 1C11 mAb.

We tested the expression of CX3CR1 in mouse tumor cell line CT26 *in vitro* and in *ex vivo* CT26 tumor harvests from tumor bearing mice and observed high expression of CX3CR1 in the tumor cells *in vitro* and *ex vivo* (**Figure 1E**). The CX3CL1-CX3CR1 axis has been shown to contribute to migration of tumor cells expressing CX3CR1 and promote metastasis (14, 26–28, 30, 32, 33, 50). We hypothesized our mAb could inhibit migration of CX3CR1+ tumor cells towards a CX3CL1 gradient. Using a Boyden chamber-based migration assay, we found that CT26 tumor cells pre-treated with CX3CR1 mAb had reduced migration towards a CX3CL1 gradient (**Figure 1F**).

To assess the clinical applicability of the 1C11 mAb, which was made in a CX3CR1 knockout mouse, we tested if the antibody would bind to human CX3CR1. We used CHO cells transfected to express human CX3CR1 (Sigma Aldrich) and observed that the 1C11 mAb could recognize and bind to human CX3CR1 with an EC-50 of 6.4 µg/ml, considerably weaker than its binding to mouse CX3CR1 (EC-50 of 0.09 µg/ml) (**Supplementary Figure 1A**).

### 3.3 Combination immunotherapy with CX3CR1 and PD-1 mAbs enhances survival in the CT26 tumor model

PD-1 monotherapy is FDA approved in 25 tumor types; however, it only works in a moderate percentage of patients as multiple resistance mechanisms limit the responsiveness of a tumor to anti-PD-1 therapy (1, 51). In non-small cell lung cancer, plasma CX3CL1 levels increased in non-responders to anti-PD-1 therapy (9). Moreover, reduced numbers of CX3CR1+CD206+ myeloid cells were associated with a positive response to anti-PD-1 therapy in a T3 sarcoma mouse model (10). Thus, we hypothesized that the CX3CL1-CX3CR1 axis could play a significant role in resistance or non-responsiveness to anti-PD-1 therapy. Hence, blockade of the CX3CL1-CX3CR1 axis might prevent resistance to anti-PD-1 immunotherapy and improve the likelihood of response in tumors with CX3CR1 expression.

**Figure 2:**
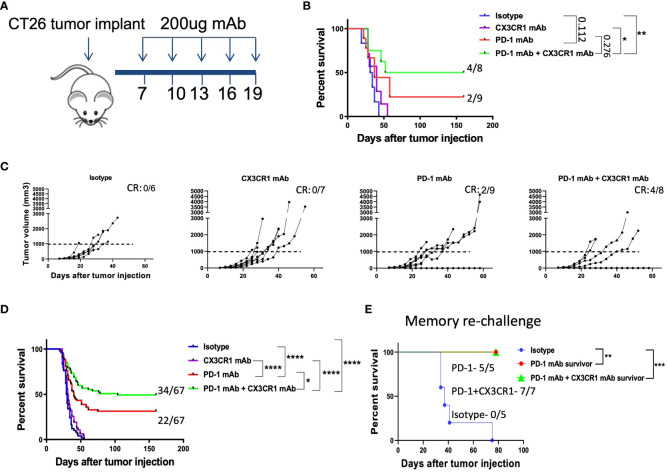
Combination immunotherapy with CX3CR1 and PD-1 mAb enhances survival in the CT26 tumor model. **(A)** Schematic of antibody treatments in the subcutaneous CT26 tumor model. BALB/cJ mice were injected with 0.2 million CT26 tumor cells on day 0 and treated with antibodies beginning on day 7. **(B, C)** Tumor growth curves and Kaplan-Meier survival curves of treatment groups: Isotype (n=6), CX3CR1 mAb (n=7), PD-1 mAb (n=9), PD-1 mAb + CX3CR1 mAb (n=8). Data of one independent experiment representative of six experiments. Numbers in upper right of **(B, C)** indicate the number of complete responders (CR). **(D)** Kaplan-Meier survival curves of treatment groups combined from six independent experiments: Isotype (n=50), CX3CR1 mAb (n=45), PD-1 mAb (n=67), PD-1 mAb + CX3CR1 mAb (n=67). **(E)** Long-term survivors from PD-1 mAb (n=5) and PD-1 mAb + CX3CR1 mAb (n=7), were re-challenged with CT26 tumor in the opposite flank and monitored for survival. Data of one independent experiment. Statistical analysis using log-rank (Mantel-Cox) test. *p<0.05, **p<0.01, ***p<0.001, ****p<0.0001.

To test our hypothesis, we chose the CT26 tumor model as CT26 expresses CX3CR1 (**Figure 1E**) and PD-L1 (**Supplementary Figures 2A, B**). We treated the CT26 tumor model with anti CX3CR1 alone and in combination with anti-PD-1 (**Figure 2A**). We observed slower tumor growth and increased survival in the anti-CX3CR1 + anti-PD-1 combination group compared to anti-PD-1 alone. (**Figures 2B, C**). Combined results of six independent experiments showed a statistically significant survival benefit between anti PD-1 + anti CX3CR1 versus anti PD-1 alone with a *p* value of 0.025 (**Figure 2D**). The percentage of long-term survivors in the combination was 51 percent while the percentage of survivors in single agent anti-PD-1 immunotherapy was 33 percent. Anti-CX3CR1 alone did not slow tumor growth.

We confirmed anti-tumor immunological memory in the survivors through a re-challenge experiment. Survivors of single agent anti-PD-1 and combined anti CX3CR1 + anti-PD-1 had 100% percent survival when re-challenged with CT26 while control mice succumbed to the tumor, confirming immunological memory after the antibody treatments (**Figure 2E**).

### 3.4 Immune suppressive myeloid cells have a high expression of CX3CR1

**Figure 3:**
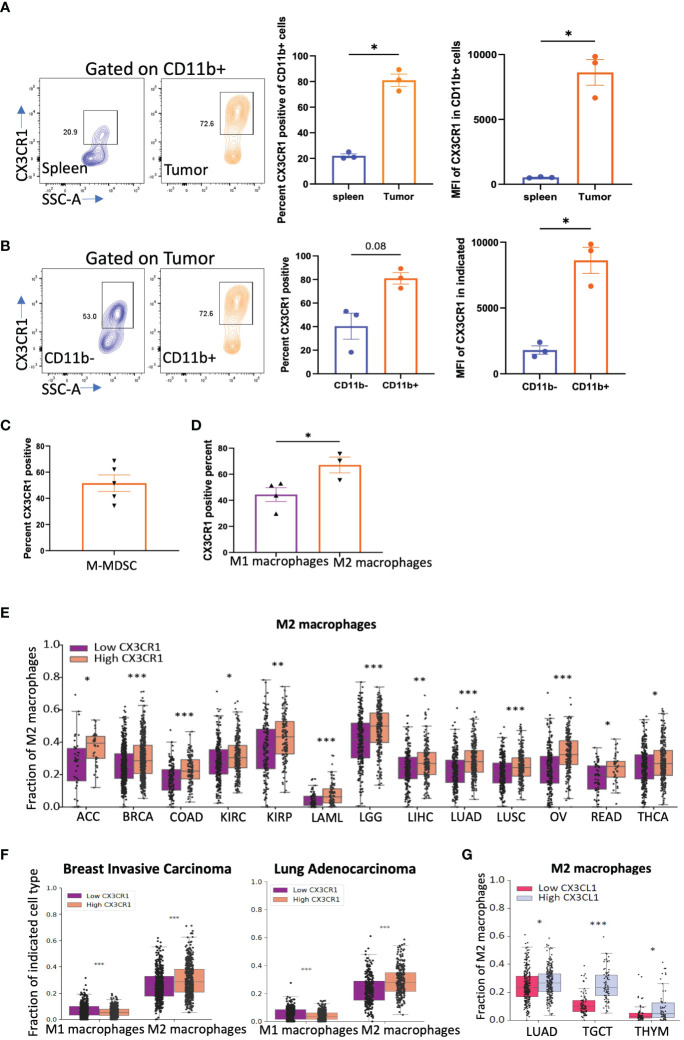
CX3CR1 expression on myeloid cells in the CT26 tumor. **(A)** FACS analysis of CX3CR1 expression on CD11b+ myeloid cells in spleen or CT26 tumors from mice (n=3) with tumor diameter of 0.5 cm. **(B)** CX3CR1 expression on CD11b+ and CD11b- cells in CT26 tumors from mice (n=3) with tumor diameter of 0.5 cm. **(C)** CX3CR1 expression in M-MDSC (CD11b+Ly6C+Ly6G-) cells in CT26 tumors from mice (n=5) with tumor diameter of 1 cm. **(D)** CX3CR1 expression on M1 macrophages (CD11b+F4/80+Gr1-MHCII+), and M2 macrophages (CD11b+F4/80+Gr1-CD206+) in CT26 tumors from mice (n=5) with tumor diameter of 1 cm. Data representative of two-three independent experiments. **(E)** Fraction of M2 macrophages in human cancers grouped as CX3CR1-high and -low by the median value of its expression and analyzed using CIBERSORT. **(F)** Fraction of M1 and M2 macrophages in human cancers grouped by high and low by the median value of CX3CR1. **(G)** Fraction of M2 macrophages in human cancers grouped by high and low by the median value of CX3CL1 expression. **(A-D)** Data as standard error of the mean, paired t-test. *p<0.05, **(E-G)** Statistical analysis using R package *limma*. *q<0.05, **q<0.01, ***q<0.001.

Tumor cells secrete factors that attract immune suppressive myeloid cells into the tumor niche (52). One important factor is CX3CL1 as myeloid cells have been shown to have high expression of CX3CR1 and traffic towards a CX3CL1 gradient found in the tumor microenvironment (50). We thus tested the expression of CX3CR1 on myeloid cells in CT26 tumors. We confirmed that CD11b+ myeloid cells in the CT26 tumor express a high percentage and MFI of CX3CR1 but those in the spleen had low expression (**Figure 3A**) consistent with previous studies. Both CD45+CD11b+ and CD45+CD3+ populations in the tumor had higher CX3CR1 expression than their corresponding populations in the spleen (**Figure 3A**, **Supplementary Figures 3A,B**). In the tumor, CX3CR1 expression was higher in CD45+CD11b+ than CD45+CD11b- cells (**Figure 3B**, **Supplementary Figure 3C**). These results indicate that CX3CR1 expression on immune cells increases in the tumor amongst which the CD11b+ myeloid cells have the highest CX3CR1 expression. The MDSC subset of myeloid cells have been shown to mediate a major resistance mechanism to anti-PD-1 therapy in colorectal cancer (4). We confirmed that a high percentage of M-MDSC in the CT26 tumor express CX3CR1 (**Figure 3C**), consistent with previous studies.

We examined other myeloid immune suppressive populations such as tumor associated macrophages (TAMs). TAMs in the CT26 tumor express CX3CR1, and within the TAM subsets, M2 macrophages had higher CX3CR1 expression than M1 macrophages (**Figure 3D**). The gating strategy used for these analyses is shown in **Supplementary Figures 3D-G**.

We next used an *in-silico* approach and grouped human cancers from TCGA based on their CX3CR1 expression into high CX3CR1 (top 50^th^ percentile) and low (bottom 50^th^ percentile) groups. Analysis of the immune populations in human tumors using CIBERSORT showed that a higher M2 macrophage fraction was associated with higher CX3CR1 expression in multiple cancer types (**Figure 3E**). In BRCA (breast carcinoma) and LUAD (lung adenocarcinoma), higher CX3CR1 expression was associated with a higher M2 macrophage fraction but a lower M1 macrophage fraction (**Figure 3F**). Moreover, higher expression of the ligand, CX3CL1, was associated with a higher M2 macrophage fraction in LUAD, TGCT (tenosynovial giant cell tumor), and THYM (thymoma) cancer types (**Figure 3G**). From these results, we hypothesized that antibody-targeting of CX3CR1 could reduce the abundance of immune suppressive myeloid populations in the tumor.

### 3.5 CX3CR1 mAb treatment can remodel myeloid cells in the CT26 tumor

**Figure 4:**
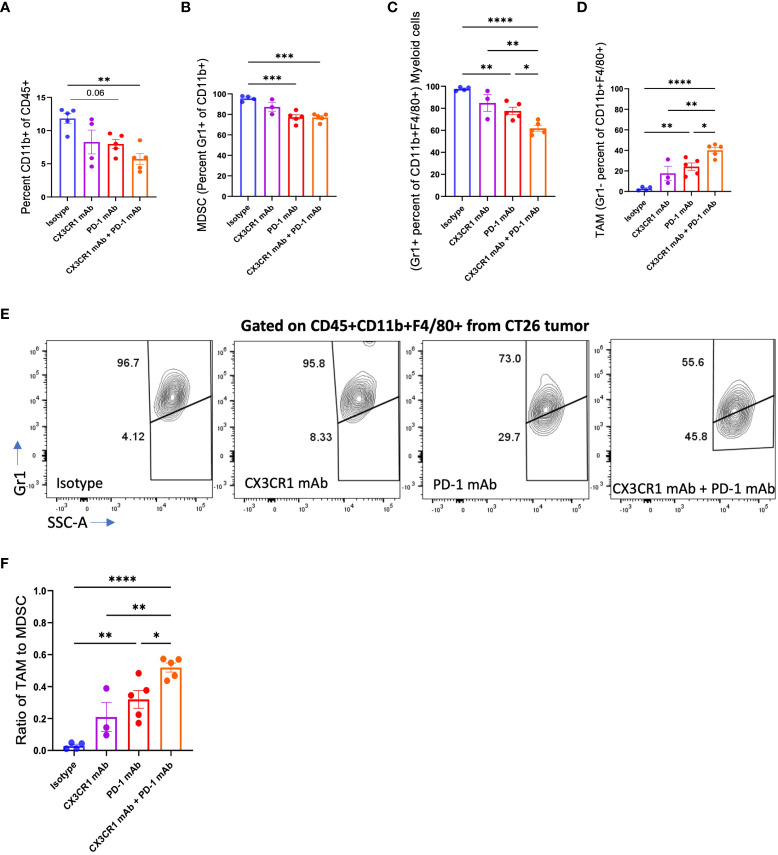
Abundance of myeloid cell subsets in CT26 tumors after mAb treatment. FACS analysis of **(A)** CD11b+, **(B)** MDSC (CD11b+Gr1+), **(C-F)** Myeloid cells (CD11b+F4/80+Gr1+) and TAM (CD11b+F4/80+Gr1-), in CT26 tumors from mice treated with the indicated antibody: Isotype (n=4-5), CX3CR1 mAb (n=3-4), PD-1 mAb (n=5), CX3CR1 mAb + PD-1 mAb (n=5). Data representative of two independent experiments. Data as one way ANOVA with Tukey’s test. *p<0.05, **p<0.01, ***p<0.001, ****p<0.0001.

We analyzed the myeloid immune subsets in the tumor microenvironment after immunotherapy treatment. Combination anti-PD-1 + anti CX3CR1 treatment led to a significantly lower percentage of CD45+CD11b+ myeloid cells in the tumor compared to isotype (**Figure 4A**). MDSC are an immune suppressive subset composed of heterogenous immature cells of myeloid origin. We classified CD11b+Gr1+ cells as MDSC based on previous work (53–55) and CD11b+ F4/80+Gr1+ cells as a sub-population of myeloid cells based on previous work by Fang et al., 2017 (56). The MDSC (CD11b+Gr1+) were the predominant CD11b+ population in the isotype-treated tumor bearing mice but were reduced in all three immunotherapy treatment groups of anti-PD-1, anti CX3CR1, and anti-PD-1 + anti CX3CR1 (**Figure 4B**). There were significantly fewer MDSC in the anti-PD-1 + anti CX3CR1 combination, and in single agent anti PD-1 therapy groups compared to the isotype. The combination anti-PD-1 + anti-CX3CR1 therapy group had the lowest percentage of CD11b+F4/80+Gr1+ myeloid cells and this was significantly less than single agent anti-PD-1 or anti-CX3CR1 groups (**Figures 4C, E**).

Conversion of immature myeloid cells to mature populations is important for a positive response to immunotherapy. Response to anti-PD-1 therapy is associated with a higher mature myeloid fraction, and a lower immature myeloid cell fraction (57). We examined the CD11b+F4/80+Gr1- myeloid cells in the tumor, representing tumor-associated macrophages (TAMs) as defined by Peranzoni et al., 2010 and Qian et al., 2010 (58, 59) and observed a significantly increased percentage in each of the immunotherapy treatment groups (**Figures 4D, E**). While the isotype treatment group had 95.6% MDSC with a negligible percent of TAM, the immunotherapy treatment groups had a lower percent of MDSC’s and a higher percentage of TAMs (**Figures 4D, E**). The combination therapy group had fewer MDSC, and significantly more TAM compared with the anti-PD-1 or anti CX3CR1 single agent groups. Importantly, the combination therapy group had a significantly increased ratio of TAMs to MDSC compared to the single agent anti-PD-1 or anti-CX3CR1 groups (**Figure 4F**). These results show the response to PD-1 mAb + CX3CR1 immunotherapy is characterized by a remodeling of the myeloid compartments in the tumor with the combination treatment skewing the myeloid populations towards more mature macrophages and fewer MDSC. Thus, the CX3CR1 antibody reduces the abundance of MDSC in the tumor. The gating strategy is shown in **Supplementary Figure 4A**.

To assess the suppressive potency of the MDSC, we analyzed expression of the CD206 marker that identifies the suppressive M2-like population of MDSC that promotes tumor growth. We observed a decreased percentage of CD206+ MDSC following treatment with single agent anti-PD-1 or combined anti-PD-1 + anti CX3CR1 immunotherapy (**Supplementary Figure 4B**).

We also investigated whether there were differences in CD8+ TILs between the antibody treatment groups since CD8 TILs are known to express CX3CR1 (60). The percentage of CD8+ TILs was not different between the treatment conditions (**Supplementary Figure 4C**). The progenitor CD8 TIL population, as defined by TCF1+PD-1+, is the population that expands in response to anti-PD-1, and some can convert to a terminally exhausted PD-1+TIM-3+ CD8+ population (60). The progenitor CD8 TIL were reported to be higher in responders to anti-PD-1 (61). We examined the PD-1+ TCF1+ and PD-1+TIM-3+ CD8 TIL populations on day 12 after two antibody treatments and saw a trend towards an increase in the PD-1+TCF1+ CD8+ cells in the CX3CR1 mAb and PD-1 mAb+ CX3CR1 mAb treatment groups compared with single agent anti-PD-1 group. The PD-1+TIM3+CD8+ population was not significantly different across the treatment groups (**Supplementary Figure 4C**). The PD-1 antibody clone RMP1-30 was used for cell surface detection of PD-1 as it binds to a different epitope on PD-1 than the treatment antibody (anti-PD-1 clone 1A12) (62).

### 3.6 CX3CL1 interaction with CX3CR1 on CT26 tumor cells promotes secretion of immune suppressive soluble mediators

CX3CR1 is a G-protein coupled receptor (GPCR) on the cell surface that initiates cell signals upon binding to its ligand, CX3CL1 (13). Moreover, the CX3CL1-CX3CR1 axis in the tumor activates multiple oncogenic programs and promotes tumorigenesis (14, 19–36).

**Figure 5:**
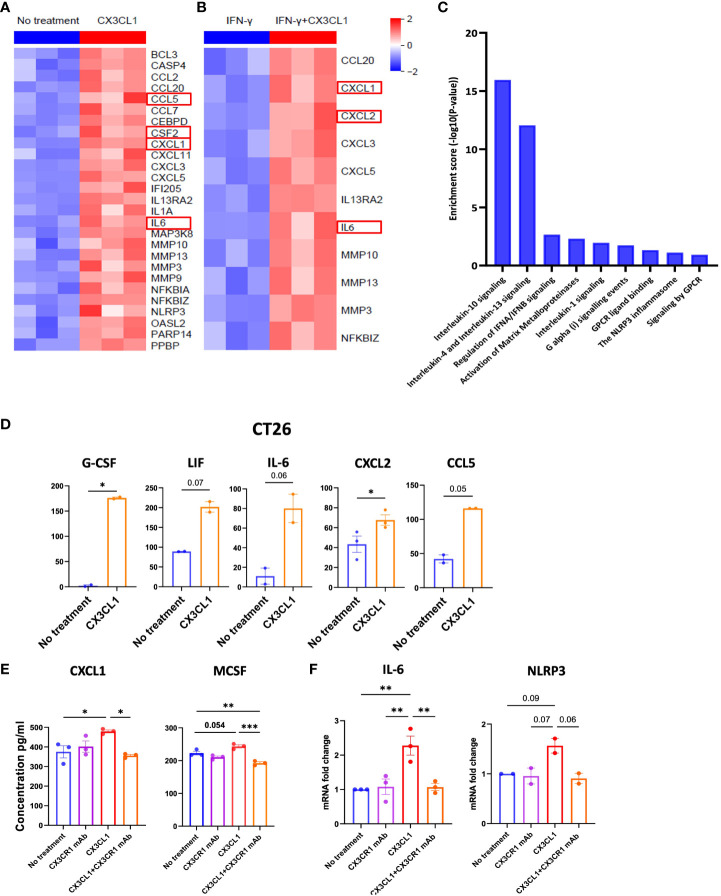
CX3CR1 mAb can reduce the secretion of immunosuppressive soluble mediators in the CT26 tumor. Heatmap of mRNA expression of CT26 tumor cell line treated with **(A)** CX3CL1 (2 µg/ml) or no treatment **(B)** IFN-γ (100 ng/ml) or IFN-γ + CX3CL1, for 8 hours (n=3). **(C)** Pathway enrichment analysis from **(A)** using Reactome database. **(D)** Quantification of G-CSF, LIF, IL-6, CXCL2 and CCL5 secreted from CT26 with CX3CL1 (2 µg/ml) for 24 hour or no treatment. Data combined from two independent experiments. Data as standard error of the mean, paired t-test. *p<0.05. **(E)** Quantification of CXCL1 and MCSF secreted from CT26 tumor cell line treated with no treatment, CX3CL1 (2 µg/ml), CX3CR1 mAb (10 µg/ml), CX3CL1 (2 µg/ml) + CX3CR1 mAb (10 µg/ml) after 24 hours. Data representative of two independent experiments. **(F)** IL-6 and NLRP3 mRNA from CT26 tumor cell line treated with no treatment, CX3CL1 (2 µg/ml), CX3CR1 mAb (10 µg/ml), CX3CL1 (2 µg/ml) + CX3CR1 mAb (10 µg/ml) after 8 hours. RNA quantified using RT-qPCR. Data combined from two-three independent experiments. Data as one way ANOVA with Tukey’s test. *p<0.05, **p<0.01, ***p<0.001.

Whether the axis can directly mediate tumor immune evasion is not known. We treated CT26 tumor cells with CX3CL1 and determined which soluble mediators were upregulated in the tumor cells by mRNA and multiplex ELISA analysis. Interestingly, production of several cytokines that promote tumor immune resistance was upregulated when CT26 tumor cells were treated with CX3CL1 (**Figures 5A, B**). This included upregulation of CXCL1, CSF-2, and IL-6 mRNA in CT26 following CX3CL1 treatment as confirmed through RNA-sequencing (**Figure 5A**). Moreover, several chemokines that are known to play a prominent role in metastasis of cancer cells, namely, CCL2, CCL5, CCL7, CCL20, CXCL1, CXCL3, CXCL5, and CXCL11 were upregulated in CT26 after treatment with CX3CL1 (**Figure 5A**). In addition, metalloproteinases MMP3, MMP9, MMP10 and MMP13, known to promote tumor growth (63), were upregulated by CX3CL1 mediated activation of CX3CR1 (**Figure 5A**). Moreover, IL-1, and NLRP3 were also upregulated, indicating development of an inflammasome signature in the tumor (**Figure 5A**). NF-kB mediates cancer development and progression, and we found that NF-kB mRNA was upregulated upon CX3CL1 addition indicating a relationship between CX3CR1 activation and NF-kB signaling (**Figure 5A**). We next tested whether CX3CL1 will induce these soluble mediators in the presence of IFN-γ, and TNF-α since these inflammatory mediators are likely to be present in the tumor microenvironment and could potentially reduce CX3CL1 induced signals. We treated CT26 tumor cells with IFN-γ, and TNF-α followed by treatment with CX3CL1 and obtained comparable results wherein CX3CL1 induced robust expression of genes that promote tumor immune-resistance and generate a pro-metastatic niche (**Figure 5B**). A pathway analysis of genes upregulated in **Figure 5A** showed enrichment of immune-suppressive pathways; IL-10 signaling, IL-13 signaling, matrix metalloproteinase and NLRP3 inflammasome in CT26 tumors following treatment with CX3CL1 (**Figure 5C**).

CX3CL1 upregulated the secretion of multiple chemoattractants involved in the trafficking and differentiation of myeloid derived suppressor cells (MDSC) in the tumor including high levels of CXCL1, G-CSF, IL-6, LIF (another member of the IL-6 family), CXCL2 (also known as macrophage inflammatory protein-2 alpha or MIP-2α), and CCL5 (also known as RANTES) (**Figure 5D**).

These results show the CX3CL1-CX3CR1 chemokine axis can upregulate mediators that attract immune suppressive myeloid populations and provide a mechanism whereby the axis can promote tumor immune evasion.

### 3.7 CX3CR1 mAb blockade can reduce the secretion of immunosuppressive soluble mediators by the CT26 tumor

We next sought to investigate the mechanisms through which the combination therapy had a higher survival benefit than single agent anti-PD-1 therapy. Since CX3CL1 signaling through CX3CR1 leads to secretion of immunosuppressive mediators by the CT26 tumor (**Figures 5A–D**), we hypothesized that CX3CR1 antibody could reduce the secretion of these mediators. Therefore, we investigated the capacity of CX3CR1 mAb to alter CX3CL1-CX3CR1 mediated effects. To test this, we pre-treated CT26 tumor cells with 10 µg/ml of the CX3CR1 mAb followed by addition of CX3CL1 recombinant protein. Pre-treatment with CX3CR1 mAb antibody resulted in a significant reduction in CXCL1 and M-CSF secretion indicating successful blockade of CX3CL1-mediated immune suppressive signals through CX3CR1 (**Figure 5E**). A reduction in IL-6 and NLRP3 mRNA was also observed (**Figure 5F**). Since tumors exploit the CX3CL1-CX3CR1 axis to make a more immune suppressive microenvironment, blocking this axis has the potential to prevent its pro-tumorigenic function.

### 3.8 Induction of CX3CL1 and CX3CR1 in CT26 by inflammatory mediators

We treated the CT26 tumor cell line with inflammatory cytokines IFN-γ and TNF-α for 24 hours and observed a 1.3-1.5-fold increase in the MFI of CX3CR1 expression compared with untreated cells (**Supplementary Figure 5A**).

The CX3CR1 ligand, CX3CL1, has been shown to be expressed on tumor cells and facilitates their migration. A unique feature of CX3CL1 as a chemokine is that it is found in membrane bound and soluble forms. The soluble form of CX3CL1 can be produced by cleavage from the membrane bound form by metalloproteinase (64, 65). Our expression analysis of CX3CL1 on mouse tumors showed that CT26 did not express CX3CL1 (**Supplementary Figure 5B**) (**Supplementary Figure 5A** and **Supplementary Table 2**).

In addition, CCL26 (Eotaxin) is a second binding partner of CX3CR1 but has 10-20-fold lower affinity than CX3CL1 (66). CCL26 is expressed only as a soluble protein. We found that CT26 did not have detectable levels of CCL26 (data not shown).

### 3.9 Expression of the CX3CL1-CX3CR1 axis in human cancers

**Figure 6:**
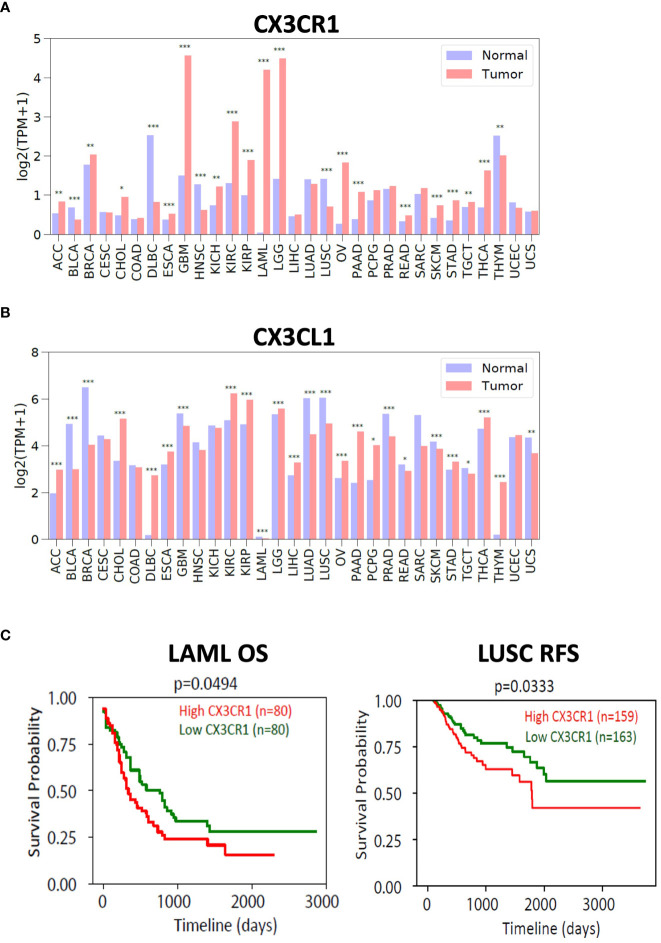
Expression of CX3CR1 and CX3CL1 in human tumors. Analysis of **(A)** CX3CR1 and **(B)** CX3CL1 expression (TPM; transcripts per million) in human tumors and normal tissue using TCGA and GTEX database. R package limma was used for statistical analysis. *p<0.05, **p<0.01, ***p<0.001. Human tumors defined as ACC (Adenoid cystic carcinoma), BLCA (Bladder urothelial carcinoma), BRCA (Breast carcinoma), CHOL (Cholangiocarcinoma), COAD (Colon adenocarcinoma), DLBC (Diffuse large B cell lymphoma), ESCA (Esophageal carcinoma), GBM (Glioblastoma), HNSC (Head and neck squamous cell carcinoma), KICH (Kidney chromophobe), KIRC (Kidney renal clear cell carcinoma), KIRP (Kidney renal papillary cell carcinoma), LAML (Acute myeloid leukemia), LGG (Low grade gliomas), LIHC (Liver hepatocellular carcinoma), LUAD (Lung adenocarcinoma), LUSC (Lung squamous cell carcinoma), OV (Ovarian carcinoma), PAAD (Pancreatic ductal adenocarcinoma), PCPG (Pheochromocytoma and paraganglioma), PRAD (Prostate adenocarcinoma), READ (Rectum adenocarcinoma), SARC (Sarcoma), SKCM (Skin cutaneous melanoma), STAD (Stomach adenocarcinoma), TGCT (Testicular germ cell tumors), THCA (Thyroid carcinoma), THYM (Thymoma), UCEC (Uterine corpus endometrial carcinoma), UCS (Uterine carcinosarcoma). **(C)** Analysis of overall survival (OS) and relapse free survival (RFS) in LAML (acute myeloid leukemia) and LUSC (lung squamous cell carcinoma). High (in red) and Low (in green) CX3CR1 based on the median value. R package survival was used for statistical analysis. P values as indicated.

To investigate the expression of the CX3CL1-CX3CR1 axis in human cancers, we used the TCGA and GTEx databases and examined the expression of CX3CR1 and CX3CL1 in human tumors versus their expression in normal tissue (**Figure 6**). We found multiple human tumor types expressed CX3CR1 with GBM (Glioblastoma), LGG (Low Grade Glioma), LAML (Acute Myeloid Leukemia), KIRC (Kidney Renal Clear Cell Carcinoma), KIRP (Kidney Renal Papillary Cell Carcinoma) having notably high expression compared with the normal tissue. CX3CL1 showed high expression in KIRC (Kidney Renal Clear Cell Carcinoma), and KIRP (Kidney Renal Papillary Cell Carcinoma). We next investigated survival in cancer patients based on CX3CR1 expression. LAML (Acute Myeloid Leukemia) and LUSC (lung squamous cell carcinoma) patient samples with high CX3CR1 expression had significantly worse overall survival and relapse free survival than those with low CX3CR1 expression (**Figure 6**).

Our results support a role for the CX3CL1-CX3CR1 axis in tumor immune evasion. Though it is well known that the axis is important in chemotaxis, its functions in immune evasion should be further explored.

## 4 Discussion

MDSC represents a major resistance mechanism that limits the response to anti-PD-1. A strategy that targets MDSC in combination with anti-PD-1 immunotherapy can improve responses (4, 5). Here, we test a novel CX3CR1 monoclonal antibody that blocks the CX3CL1-CX3CR1 interaction and promotes responses to anti-PD-1 immunotherapy in a mouse tumor model. Our results show that the response to PD-1 mAb + CX3CR1 immunotherapy is characterized by a remodeling of the myeloid compartments in the tumor with the combination treatment skewing the myeloid populations towards fewer MDSC and more mature macrophages. Our data suggest that the axis can be involved in the MDSC-mediated mechanism of resistance to anti-PD-1 immunotherapy.

We observe higher survival and response in the syngeneic immunocompetent CT26 tumor model when anti-CX3CR1 is combined with anti-PD-1. Both anti-PD-1 and anti-PD-1 + anti-CX3CR1 long-term survivor mice show immunological memory. Generation of effector memory may be the central mediator of immunological memory based on previous studies (67). CX3CR1 is known to promote migration of CX3CR1+ tumor cells (14–18) and our anti-CX3CR1 antibody reduces the migration of CX3CR1+ CT26 colorectal carcinoma. In addition, the CX3CR1 mAb reduces secretion of soluble mediators from the tumor and reduces the abundance of immune suppressive myeloid cell populations while increasing the percentage of mature macrophages. Treatment with CX3CR1 mAb also resulted in a reduction of NLRP3 mRNA, a component of the tumor inflammasome. Previous studies have shown that NLRP3 inhibition (68) reduces MDSC infiltration into the tumor suggesting an additional mechanism for how CX3CR1 mAb blockade can reduce tumor immune evasion.

The CX3CL1-CX3CR1 axis activates multiple oncogenic programs in the tumor including PI3K/Akt, ERK, EGFR, p38, β-integrin, MMP2/9/14, ICAM, VCAM, JAK/STAT, MAPK, ICAM and p38 (14, 19–36). Our results show that this axis generates a pro-tumorigenic and immunosuppressive program as MAPK38, PARP, NLRP3, MMP3/9/10/13, NF-kB, IL-1 are upregulated and multiple soluble mediators including G-CSF, CCL2, CCL5 (alias; RANTES), CXCL7, CCL20 (alias; macrophage inflammatory protein-3; MIP-3α), CXCL1, CXCL3, CXCL5, CXCL11, IL-6, LIF (member of the IL-6 family) are secreted when CX3CR1 positive CT26 tumor cells are treated with CX3CL1 recombinant protein. These factors are known to drive the migration of immune suppressive myeloid cells (MDSC) into tumor (6). Thus, we show how the CX3CL1-CX3CR1 axis in CX3CR1+ tumors can be responsible for inducing the secretion of soluble factors that recruit and maintain MDSC. We further show that M-MDSC and M2 macrophages have high expression of CX3CR1, consistent with previous reports (69, 70).

Secretion of soluble CX3CL1 can be part of a feedback loop to maintain a CX3CR1 driven immune-suppressive signaling program in the tumor. Adaptive resistance to immune attack can be mediated by tumor cells increased expression of PD-L1 on their cell surface in response to IFN-γ. CX3CL1 expression is also known to increase in response to IFN-γ and TNF-α (71) due to STAT1 and NF-kB response elements in the CX3CL1 promoter. Our study confirmed that IFN-γ and TNF-α inflammatory mediators can increase CX3CR1 cell surface expression in CT26 but did not upregulate CX3CL1 in CT26. Activated T cells can also express CX3CR1 (72) and require cell to cell contact with the tumor to mediate tumor cell lysis. Indeed, high serum concentrations of soluble CX3CL1 were associated with low T cell cytotoxicity while low serum concentrations of soluble CX3CL1 were associated with high T cell cytotoxicity at the tumor site in a mouse model of adoptive T cell therapy of colon carcinoma (73).

Small molecule antagonists to CX3CR1 such as JMS-17-2 bind to both CX3CR1 and CCR1 (34). Thus, a limitation of small molecule CX3CR1 antagonists is that they are not specific to CX3CR1 and can bind to additional members of the GPCR family. In addition, toxicities due to non-specific binding of the antagonist can occur. In addition, these antagonists do not show selective bias for cells with higher target expression unlike an antibody-mediated effect. Moreover, there is often promiscuity in binding of chemokines to chemokine receptors. For example, chemokine CXCL12 binds to both CXCR4 and CXCR7 in glioblastoma (28, 74). Since tumors can co-express multiple chemokine receptors (28, 52, 74, 75), blockade of one receptor-ligand axis could still allow function through a second receptor-ligand axis to promote tumorigenic effects. Thus, an important advantage of targeting CX3CR1 is that CX3CR1 is the only binding receptor for CX3CL1 (76). As our knowledge deepens of the important cell types involved in the anti-tumor response mediated by anti-CX3CR1 mAb, an adaptation of the mAb design into a bi-specific to specifically target myeloid or other immune suppressive populations could be investigated. For successful translation to human, a fully human or humanized anti-human CX3CR1 mAb would need to be generated in order to avoid anti-antibody effects on therapeutic efficacy.

In summary, our results show that the CX3CL1-CX3CR1 axis can promote tumorigenesis and immune-suppression by 1) Signaling through CX3CR1 that recruits myeloid populations and enhances secretion of soluble mediators by the tumor; 2) Increase of NLRP3 inflammasome component in the tumor; 3) Increase of pro-tumorigenic programs in the tumor. By reducing these activities, the CX3CR1 monoclonal antibody can disrupt recruitment, facilitate remodeling, and alter the MDSC compartment in the tumor microenvironment (**Supplementary Figure 6**), making the combination of CX3CR1 and PD-1 antibody blockade a promising combination therapeutic strategy. Since CX3CR1 is expressed on multiple cancer types, CX3CR1 antibody blockade could show therapeutic efficacy in multiple cancers.

## Data availability statement

The datasets presented in this study can be found in online repositories. The names of the repository/repositories and accession number(s) can be found below: https://www.ncbi.nlm.nih.gov/geo/, GSE215269.

## Ethics statement

The animal study was approved by Dana-Farber Cancer Institute Institutional Animal Care and Use Committee. The study was conducted in accordance with the local legislation and institutional requirements.

## Author contributions

AC and GF: Conception and design. AC, XB, YW, MG, PHu, and S-HH: Data acquisition. AC, YW, and MG: analyzed the data and produced the figures. AC, XB, YW, MG, JT, GL, MD, UvA, PHw, and GF contributed to the interpretation of the data. AC and GF wrote the manuscript. AC, XB, YW, MG, PHu, S-HH, GL, MD, UvA, PHw, and GF edited the manuscript. All authors contributed to the article and approved the submitted version.
