# Supplementary material for: The CX3CL1-CX3CR1 chemokine axis can contribute to tumor immune evasion and blockade with a novel CX3CR1 monoclonal antibody enhances response to anti-PD-1 immunotherapy
Source: Front Immunol. 2023 Sep 13;14:1237715. doi: 10.3389/fimmu.2023.1237715 (PMC10524267; doi:10.3389/fimmu.2023.1237715)
Supplement: Supplementary file 1 [file DataSheet_1.pdf]

Antibody	Catalog	Clone	Company	RRID
Live/dead	L34964		eBioscience	
CD45	cat 103114	30-F11	Biolegend	RRID:AB_312979
CD11b	cat 101217	M1/70	Biolegend	RRID:AB_389305
Ly6C	cat 128016	HK1.4	Biolegend	RRID:AB_1732076
Ly6C	cat 128043	HK1.4	Biolegend	RRID:AB_2566576
Ly6G	cat 563978	1A8	BD Biosciences	RRID:AB_2716852
Ly6G	cat 127621	1A8	Biolegend	RRID:AB_10640452
F4/80	cat 123130	BM8	Biolegend	RRID:AB_2293450
CD206	cat 141708	C068C2	Biolegend	RRID:AB_10900231
Gr1	cat 108452	RB6-8C5	Biolegend	RRID:AB_2564249
NOS	ref 17-5920-82	CXNFT	eBioscience	RRID:AB_2573244
MHCII	cat 743876	2G9	BD Biosciences	RRID:AB_2741827
CD3	cat 100307	145.2C11	Biolegend	RRID:AB_312672
CD8	cat 566409	53-6.7	BD Biosciences	RRID:AB_2744467
PD1	cat 566876	RMP1-30	BD Biosciences	RRID:AB_2869928
TCF1	cat 6444S	C63D9	Cell signaling technology	RRID:AB_2797627
Tim3	cat 747620	5D12	BD Biosciences	RRID:AB_2744186
CX3CR1	Freeman laboratory	8H12		
CX3CR1	Freeman laboratory	1C11		
PD-1	cat BEO273	1A12	BioXcell	RRID: AB_2687796

Supplementary Table 1. Fluorochrome conjugated antibodies

A

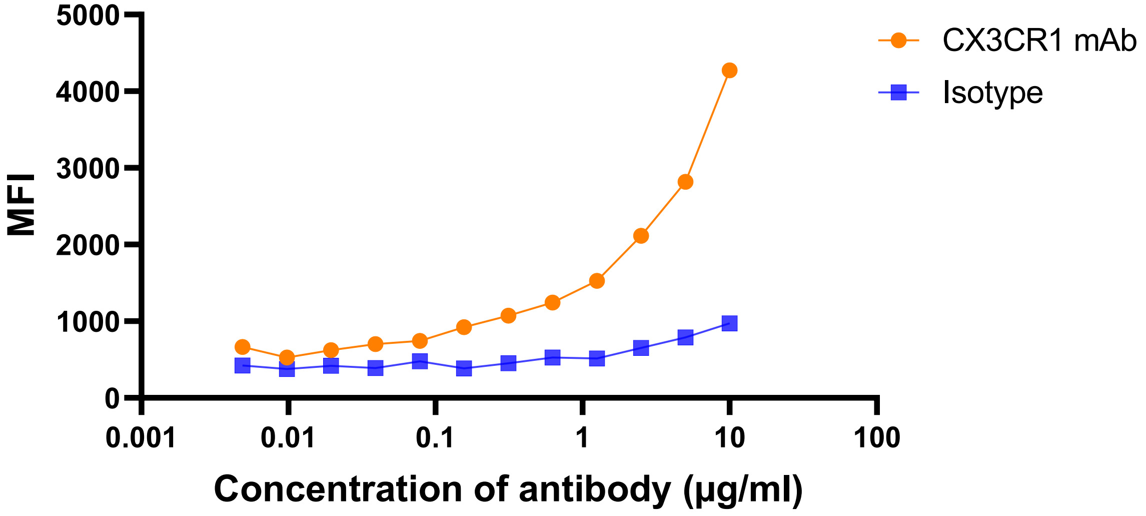

Supplementary Figure 1. CX3CR1 mAb clone 1C11 recognizes human CX3CR1
A. FACS analysis of CX3CR1 mAb binding to CHO cells stably expressing human CX3CR1.

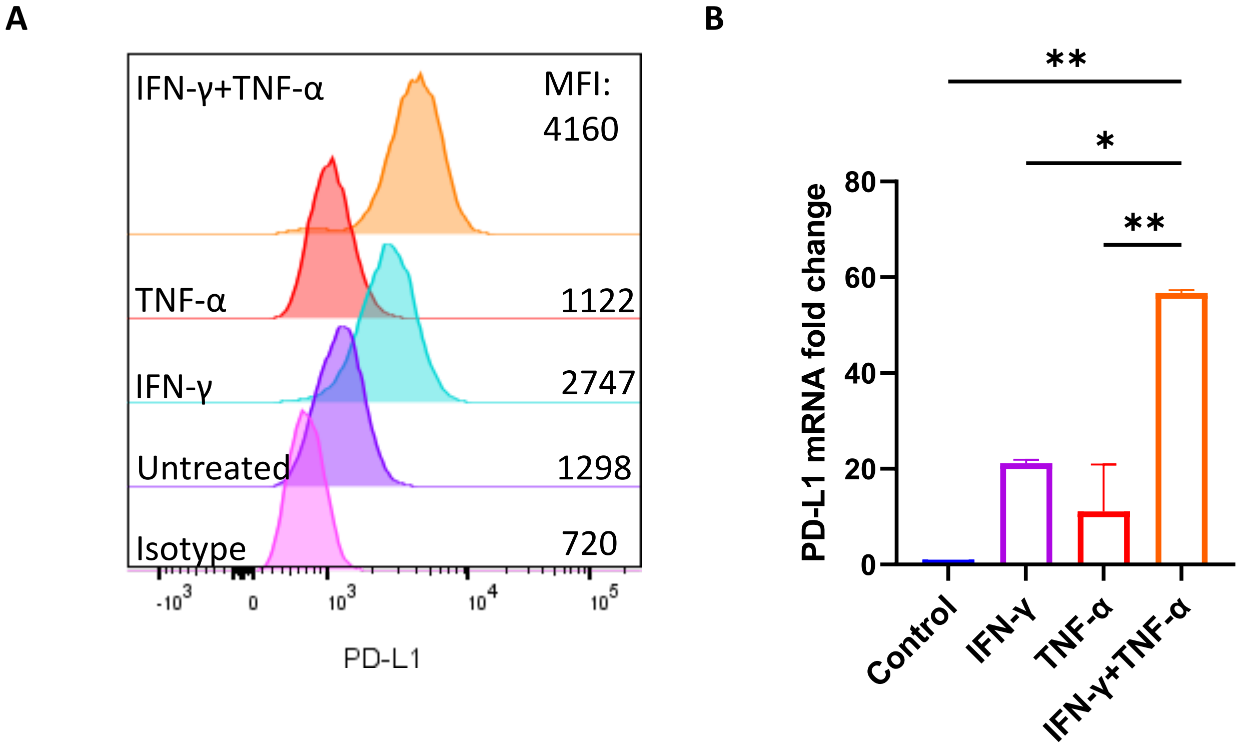

Supplementary Figure 2. Expression of PD-L1 in CT26 tumor

CT26 tumor cells were untreated or treated with IFN-γ (100 ng/ml), TNF-α (100 ng/ml), IFN-γ + TNF-α and analyzed at 24 hrs by FACS or 8 hrs for mRNA expression. **A.** PD-L1 cell surface expression. Data representative of three independent experiments. **B.** PD-L1 mRNA was quantified using RT-qPCR. Data combined from two independent experiments. Data as standard error of the mean, one way ANOVA with Tukey's multi-comparison test. *p<0.05, **p<0.01.

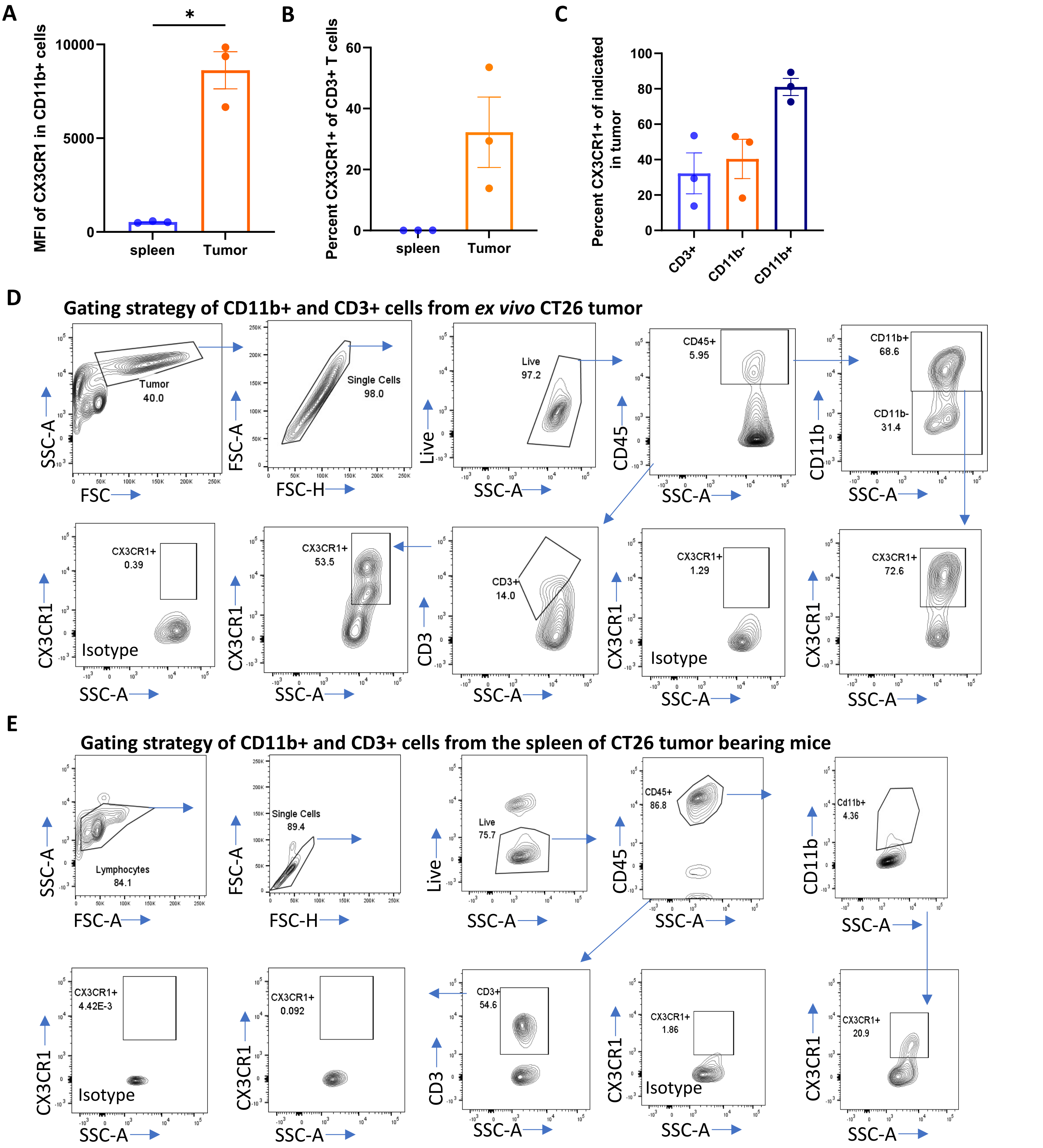

Supplementary Figure 3. CX3CR1 expression in CD11b+ myeloid cells and CD3+ T cells in CT26 tumor

A. FACS analysis of CX3CR1 expression in CD11b+ myeloid cells in spleen or CT26 tumors from mice (n=3). **B.** FACS analysis of CX3CR1 expression in CD3+ T cells in spleen or CT26 tumors from mice (n=3). **C.** FACS analysis of CX3CR1 expression in CD3+, CD11b-, and CD11b+ cells in CT26 tumors from mice (n=3). Data representative of two independent experiments. Data as standard error of the mean, paired t-test. *$p<0.05$. **D. and E**. Gating strategy of tumor and spleen CD11b+ and CD3+ populations.

F

Gating strategy of M-MDSC from *ex vivo* CT26 tumor

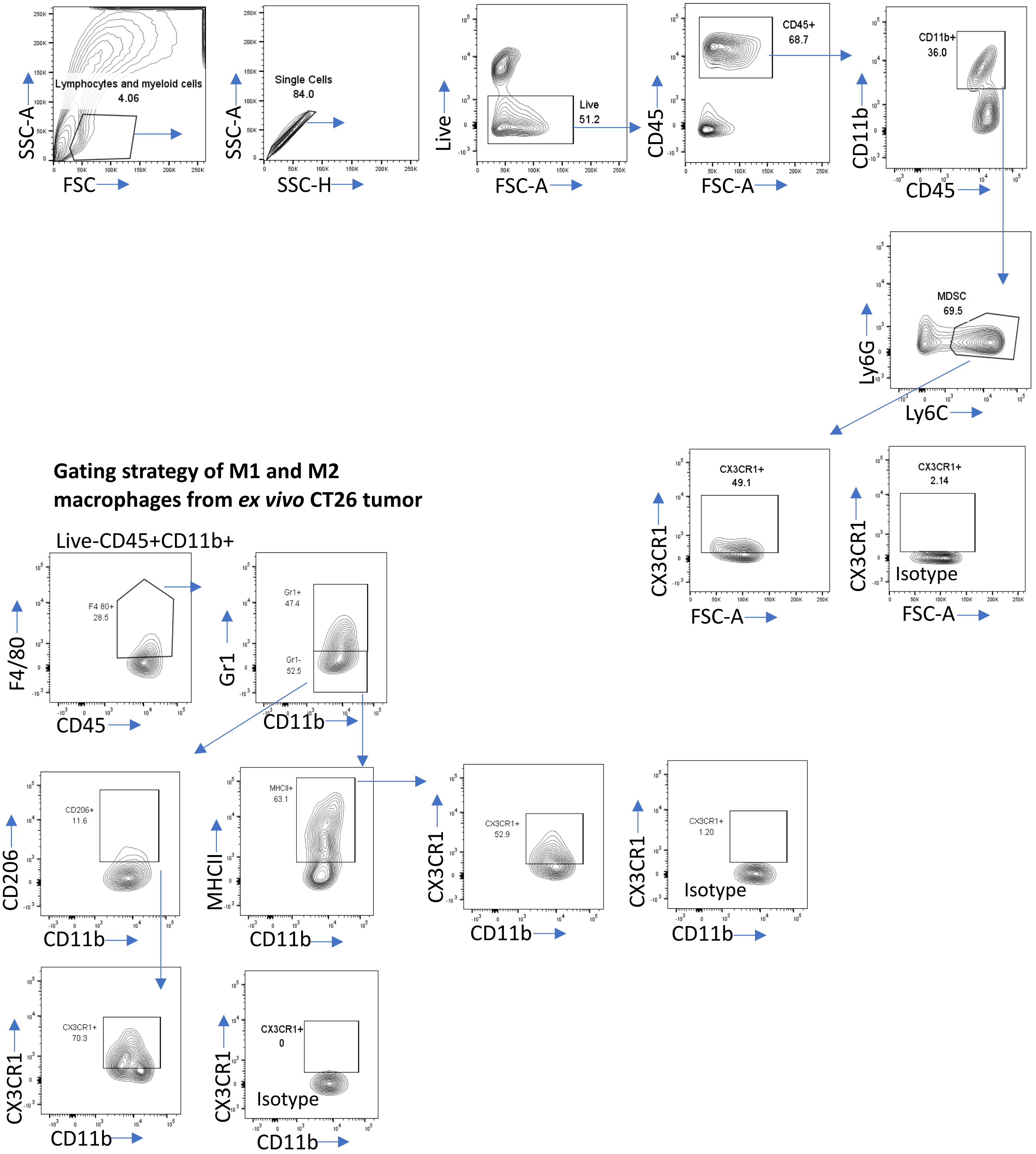

Supplementary Figure 3. continued

F. Gating strategy of M-MDSC from CT26 tumor. **G**. Gating strategy of M1 and M2 macrophages from CT26 tumor.

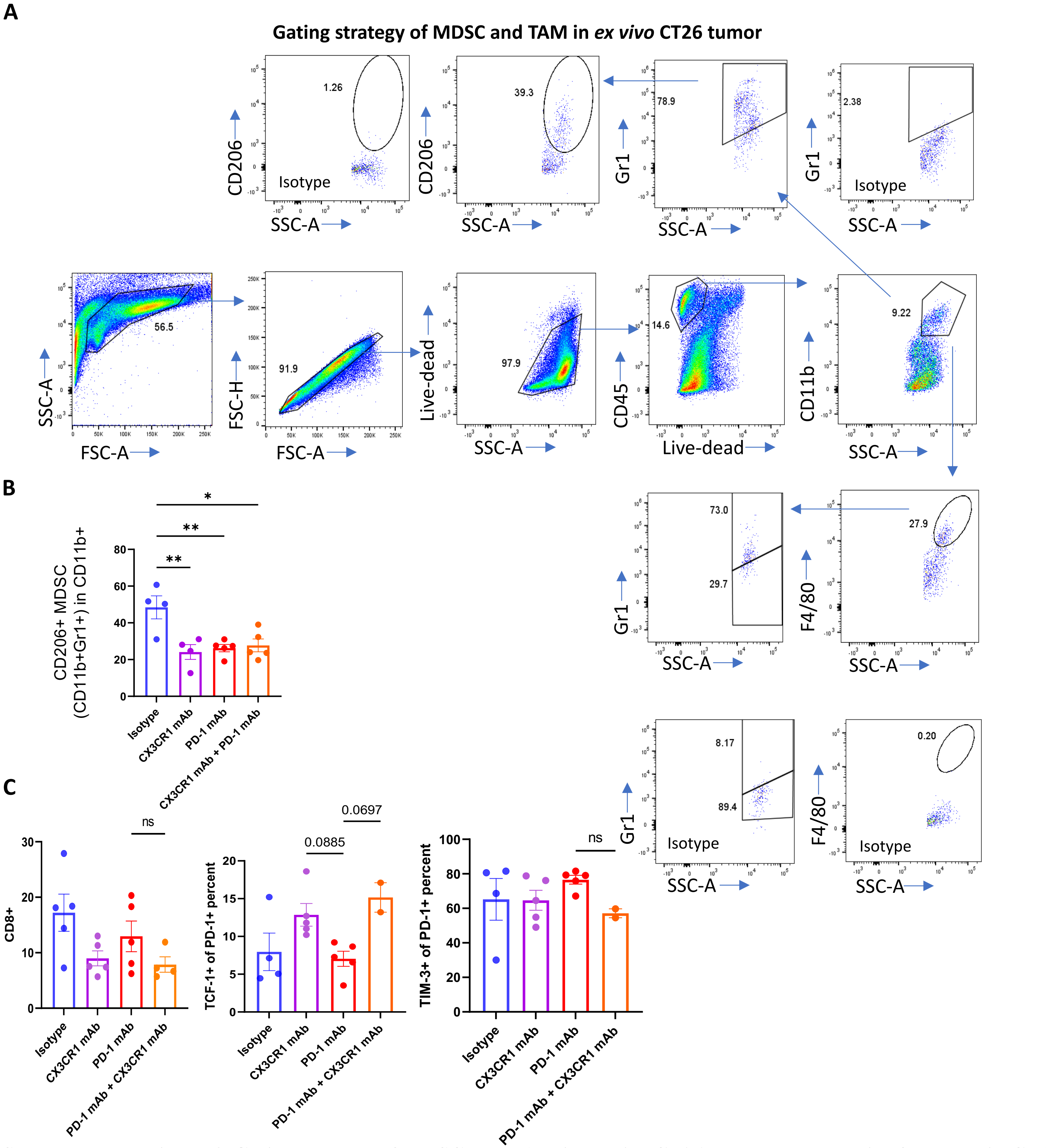

Supplementary Figure 4. Gating strategy of MDSC and TAM in *ex vivo* CT26 tumor and analysis of changes in CD8 T cell populations

A. Gating strategy of MDSC and TAM in CT26 tumor bearing mice. **B.** FACS analysis of CD206+ MDSC (CD11b+Gr1+) in CT26 tumors from mice treated with the indicated antibody: Isotype (n=4-5), CX3CR1 mAb (n=3-4), PD-1 mAb (n=5), CX3CR1 mAb + PD-1 mAb (n=5). Data representative of one independent experiment. **C.** FACS analysis of CD8+, PD-1+TCF1+CD8+, PD-1+TIM3+CD8+ T cells in CT26 tumors from mice treated with the indicated antibody: Isotype (n=4-5), CX3CR1 mAb (n=5), PD-1 mAb (n=5), CX3CR1 mAb + PD-1 mAb (n=2-4). Data representative of two independent experiments. Data as one way ANOVA with Tukey's test. *p<0.05, **p<0.01.

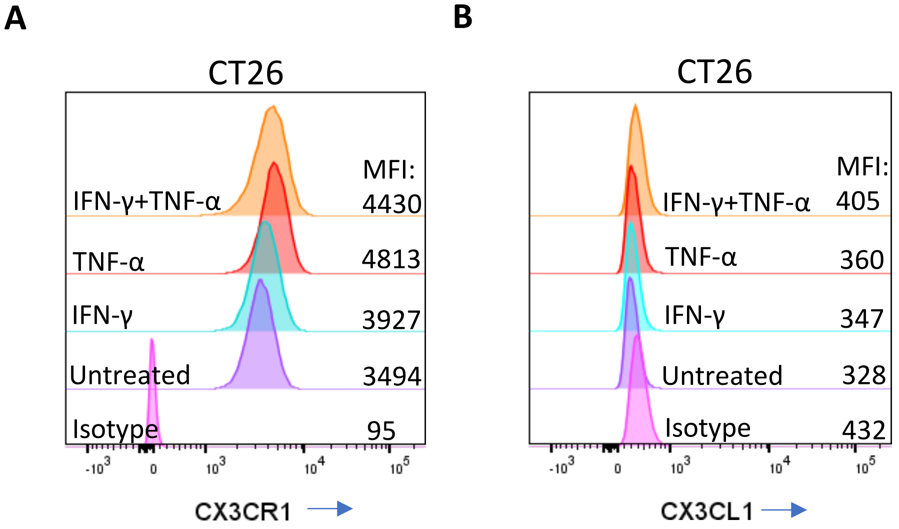

Supplementary Figure 5. Expression of CX3CL1-CX3CR1 chemokine axis in mouse and human tumor cells and its induction through inflammatory mediators

A and B. FACS analysis of CX3CR1 and CX3CL1 expression on CT26 cell line untreated or treated with: IFN-γ (100 ng/ml), TNF-α (100 ng/ml), IFN-γ + TNF-α for 24 hrs. Data representative of three-four independent experiments.

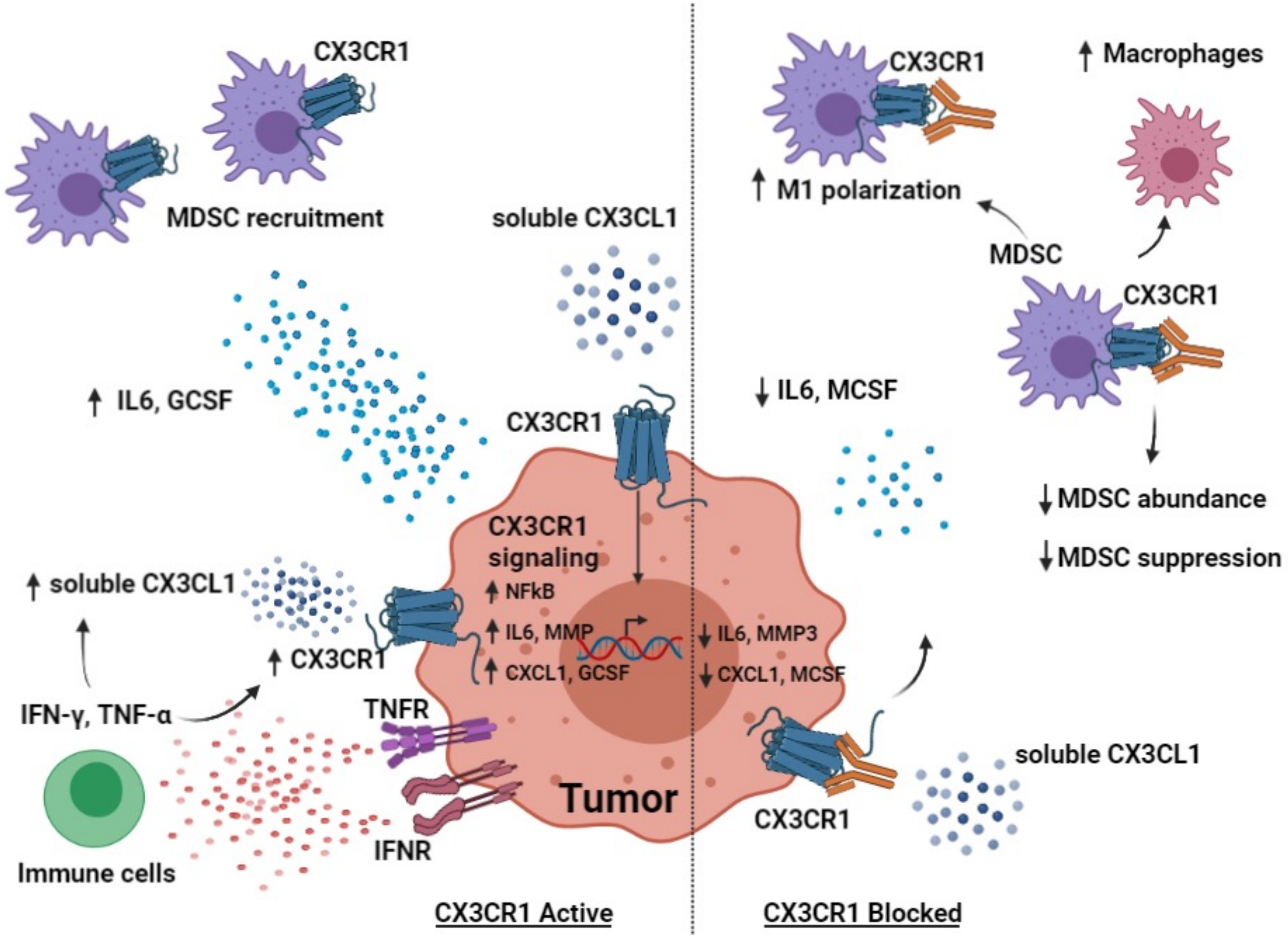

Supplementary Figure 6. The CX3CL1-CX3CR1 chemokine axis can contribute to tumor immune evasion and blockade with a novel CX3CR1 monoclonal antibody enhances responses to anti-PD-1 immunotherapy

Supplementary Analysis 1. *In silico* prediction of interactions between mCX3CL1 and mCX3CR1 and between the 1C11 mAb and mCX3CR1 for the identification of residues in mCX3CR1 that make contact with both mCX3CL1 and the 1C11 mAb.

RESULTS

Computational Prediction of hCX3CR1 and hCX3CL1 Binding Complex

To model the binding interactions between human CX3CR1 and human CX3CL1, we used the amino acid sequences from RCSB PDB Accession Code 7XBW deposited by Lu et. al[1], who determined the crystal structure of the human CX3CL1-CX3CR1-G_{i1} complex. After modifying the amino acid sequence from the PDB file by removing linkers as described below, we inputted the amino acid sequences of hCX3CR1 and hCX3CL1 into the alphafold prediction tool of the ChimeraX[2] program, which runs the sequences through the AlphaFold2-Multimer[3] implementation in ColabFold[4]. The predicted complex of hCX3CR1-hCX3CL1 with the highest confidence was then visualized in the ChimeraX program. The predicted complex had a pTM score of 0.88 and an ipTM of 0.89, indicating a high confidence model. Similar to the crystal structure determined by Lu et. al[1], in the predicted model, the hCX3CL1 globular domain interacts with the hCX3CR1 N-terminus and the hCX3CL1 N-terminus is nested inside the helical bundle.

Computational Prediction of mCX3CR1 and mCX3CL1 Binding Interfaces

To model the binding interactions between mouse CX3CR1 and mouse CX3CL1, we obtained the mCX3CR1 amino acid sequence from UniProtKB[5] Accession Number Q9Z0D9 and the mCX3CL1 amino acid sequence from Novus Biologicals Catalog #NBP2-35038 Product Datasheet[6]. The mCX3CR1 amino acid sequence was then aligned with the inputted hCX3CR1 amino acid sequence using the Clustal Omega Multiple Sequence Alignment tool[7] and shortened to match the alignment with the hCX3CR1 sequence. We inputted the sequences of modified mCX3CR1 and mCX3CL1 into the alphafold prediction tool of the ChimeraX program. The predicted complex of mCX3CR1-mCX3CL1 with the highest confidence was then visualized in the ChimeraX program. The predicted complex had a pTM score of 0.88 and an ipTM of 0.88, indicating a high confidence model. Similar to the predicted hCX3CR1-hCX3CL1 structure, the mCX3CL1 globular domain interacts with the mCX3CR1 N-terminus and the mCX3CR1 N-terminus is nested inside the helical bundle.

To better establish the binding interface, we used the ChimeraX contacts tool to predict the residues that participate in the interaction between mCX3CR1 and mCX3CL1. Two analyses were conducted to select for the residues most likely in contact. The first analysis identified residues with buried solvent-accessible surface area greater than 15.0 Å and are displayed in Table 1. The second analysis identified residues on mCX3CR1 and mCX3CL1 with atomic

distance less than 3.5 Å from mCX3CL1 or mCX3CR1, respectively, and the results are displayed in Table 2. Residues that were identified by both analyses are shown in Table 3.

Our contact analysis shows a total of 24 residues within mCX3CR1 that contribute to the binding interaction with mCX3CL1, and they were classified in Table 4. 14 residues were identified within the N-terminus domain, 1 residue was identified within ExtraCellular Loop 1 (ECL1), 5 residues were identified within ECL2, 3 residues were identified within ECL3, and 1 residue was identified within TransMembrane Domain VII (TMD VII). These results suggest that the binding interface on mCX3CR1 is mostly formed by the N-terminus domain with minor contributions from ECL2 and ECL3. The other ECL and TMD contribute little to the binding interaction.

Computational Prediction of 1C11 and mCX3CR1 Binding Interfaces

To model the binding interactions between the 1C11 mAb and mCX3CR1, we inputted the truncated amino acid sequence of mCX3CR1 and the variable region sequences of the 1C11 mAb into the alphafold prediction tool of the ChimeraX program. The predicted model of 1C11 mAb-mCX3CR1 with the highest confidence was visualized in the ChimeraX program.

The binding complex prediction suggests that the 1C11 mAb heavy and light chains adopt the conventional immunoglobulin domain topology with the antiparallel β-sheets being held together by intramolecular disulfide bonds (V_H: Cys22:Cys98, V_L: Cys23:Cys94). They also suggest that 1C11 mAb binds to the extracellular loops of mCX3CR1, which would allow for competitive inhibition of mCX3CL1-mediated activation of mCX3CR1.The predicted model had a pTM score of 0.54 and ipTM score of 0.31. The lower pTM and ipTM scores of the 1C11 mAb-mCX3CR1 model compared to those of hCX3CR1-hCX3CL1 and mCX3CR1-mCX3CL1 are likely due to the novelty of both the 1C11 mAb and the interaction between the 1C11 mAb and mCX3CR1. We believe that this model can give tentative insight into the structural biology of the 1C11 mAb - mCX3CR1 binding interface.

To establish the binding interface, we used the ChimeraX contacts tool to predict the residues that participate in the interaction between mCX3CR1 and the 1C11 mAb. Two analyses were conducted to select for the residues most likely in contact. The first analysis identified residues with buried solvent-accessible surface area greater than 15.0 Å and are displayed in Table 5. The second analysis identified residues on mCX3CR1 and the 1C11 mAb V regions with atomic distance less than 3.5 Å from the 1C11 mAb V regions or mCX3CR1, respectively, and the results are displayed in Table 6. Residues that were identified by both analyses are shown in Table 7 and 8.

Our contact analysis shows a total of 20 residues within the V_H region of the 1C11 mAb that contribute to the binding interaction. Of these, 2 residues were identified within the CDR-H1 loop, 10 residues were identified within the CDR-H2 loop, 4 residues were identified within the

CDR-H3 loop, and 5 residues were identified within non-CDR regions. The V_H region residue distribution suggests that all three CDRs of the V_H region contribute to the interaction with mCX3CR1, however, CDR-H2 appears to have the most engagement with mCX3CR1. Our contact analysis also shows a total of 7 residues within the V_L region of the 1C11 mAb that contribute to the binding interaction. Of these, 5 residues were identified within the CDR-L1 loop and 2 residues were identified within the CDR-L3 loop. The V_L region residue distribution suggests that CDR-L1 and CDR-L3 contribute to the binding interaction, and CDR-L2 appears to not engage mCX3CR1. The number of predicted contacts of the 1C11 mAb V_H region is greater than that of the 1C11 mAb V_L region, suggesting that the V_H region contributes the most to the binding capabilities of the 1C11 mAb.

Our contact analysis shows a total of 16 residues in mCX3CR1 that contribute only to the binding interaction with the 1C11 V_H and a total of 8 residues that contribute only to the binding interaction with the 1C11 V_L. D173 interacts with both the 1C11 V_H and V_L regions, suggesting its importance in the binding interaction. Of the combined 25 residues, 9 residues were identified within the N-terminus domain, 8 residues were identified within ECL2, 3 residues were identified within ECL3, and 5 residues were identified within ECL1. These results suggest that the binding interface on mCX3CR1 is mostly formed by the N-terminus domain and ECL2 with minor contributions from ECL1 and ECL3.

Residues in mCX3CR1 that were found in the contact analysis with the 1C11 mAb and with mCX3CL1 are displayed in Table 9. A total of 10 residues in mCX3CR1 were found in both analyses; 7 were identified within the N-terminus domain, 2 were identified within ECL2, and 1 was identified within ECL3. This suggests that the 1C11 mAb competitively binds to the N-terminus domain and blocks mCX3CL1 interactions with the N-terminus. Furthermore, through engagement of the extracellular loops, the 1C11 mAb spatially blocks mCX3CL1 from inserting into the helical bundle pocket. In conclusion, through *in silico* analysis based on the known co-crystal structure of hCX3CL1 in complex with hCX3CR1, we predicted interactions between mCX3CL1 and mCX3CR1 and between the 1C11 mAb and mCX3CR1 (Figure SA1). This analysis identified 10 residues in mCX3CR1 that make contact with both mCX3CL1 and the 1C11 mAb. These findings are consistent with the experimentally determined blockade of mCX3CL1 binding to mCX3CR1 by the 1C11 mAb.

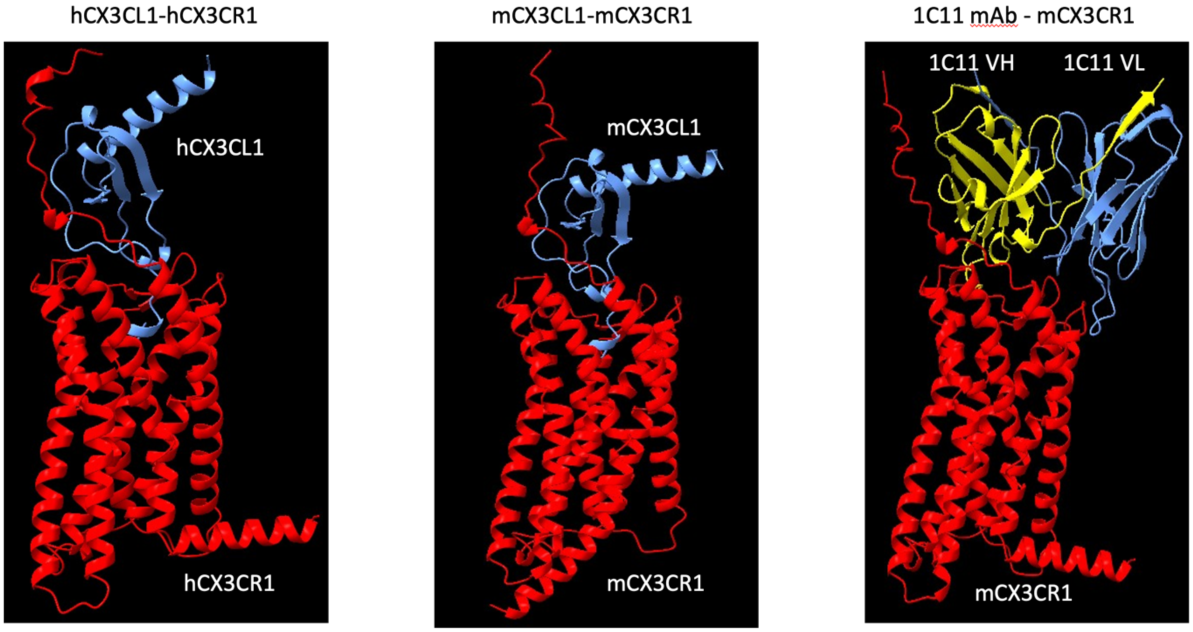

Figure SA1. Predicted interactions between mCX3CL1 and mCX3CR1 and between the 1C11 mAb and mCX3CR1.

**Contacts of Interest**	**Residues**
Residues of mCX3CR1 in contact with mCX3CL1	L8, L10, F13, Y15, D16, D17, S18, A19, E20, A21, C22, Y23, L24, G25, V28, W88, Y91, L92, F110, F111, K172, D173, E175, L177, R192, Y248, K258, K269, R273, L276, E280
Residues of mCX3CL1 in contact with mCX3CR1	Q1, H2, L3, G4, M5, T6, K7, C8, E9, I10, M11, K14, T16, I19, P20, A22, L23, E32, S33, Q45, R47, R48, F49
**Table 1:** Residues Identified by Buried Solvent-Accessible Surface Area Analysis of mCX3CR1-mCX3CL1	

**Contacts of Interest**	**Residues**
Residues of mCX3CR1 in contact with mCX3CL1	L8, F13, Y15, D16, D17, S18, A19, E20, A21, C22, Y23, L24, G25, V28, L35, Y39, Y91, S94, K172, D173, E175, C176, L177, R192, K269, R273, L276, E280
Residues of mCX3CL1 in	Q1, H2, L3, G4, M5, T6, K7, C8, E9, M11, K14, P20, Q31,

contact with mCX3CR1	E32, S33, Q45, R47, R48, F49
**Table 2:** Residues Identified by Atomic Distance Analysis of mCX3CR1-mCX3CL1	

Contacts of Interest	Residues
Residues of mCX3CR1 in contact with mCX3CL1	L8, F13, Y15, D16, D17, S18, A19, E20, A21, C22, Y23, L24, G25, V28, Y91, K172, D173, E175, L177, R192, K269, R273, L276, E280
Residues of mCX3CL1 in contact with mCX3CR1	Q1, H2, L3, G4, M5, T6, K7, C8, E9, M11, K14, P20, E32, S33, Q45, R47, R48, F49
**Table 3:** Residues Identified by Both Analyses of mCX3CR1-mCX3CL1	

mCX3CR1 Region	Residues
N-terminus	L8, F13, Y15, D16, D17, S18, A19, E20, A21, C22, Y23, L24, G25, V28
ECL1	Y91
ECL2	K172, D173, E175, L177, R192
ECL3	K269, R273, L276
TMD VII	E280
**Table 4:** Classification of mCX3CR1 Contact Residues with mCX3CL1	

Contacts of Interest	Residues
Residues in V_H region in contact with mCX3CR1	S17, S30, N31, Y32, F53, K54, S55, K57, F58, A60, N61, Y62, K67, G68, A71, D76, E84, S86, D101, P105, F106, Y108
Residues in V_L region in contact with mCX3CR1	Y31, S32, G33, N34, K36, W56, R96, Y98, G99, Y100
Residues in mCX3CR1 in	L8, L10, F13, Y15, D17, A19, E20, A21, Y23, K172, D173,

contact with V_H	L177, D179, Y180, E182, Q185, E186, K258, F259, K269, R273
Residues in mCX3CR1 in contact with V_L	Y23, D26, V28, T32, L92, I93, S94, H95, E96, D173, N174, E175

Table 5: Residues Identified by Buried Solvent-Accessible Surface Area Analysis of 1C11-mCX3CR1

Contacts of Interest	Residues
Residues in V_H region in contact with mCX3CR1	S17, S30, N31, Y32, V50, K54, S55, K57, F58, A60, N61, Y62, E64, V66, K67, G68, R69, F70, A71, D76, M85, S86, D101, P105, F106, Y108
Residues in V_L region in contact with mCX3CR1	Y31, S32, G33, N34, K36, Y38, Y98, Y100
Residues in mCX3CR1 in contact with V_H	L10, F13, Y15, S18, A19, E20, A21, C22, Y23, L24, G25, K172, D173, D179, Y180, E182, Q185, E186, P189, N193, E255, T256, K258, F259, C266, K269, R270, R273
Residues in mCX3CR1 in contact with V_L	V28, T32, L92, I93, S94, H95, E96, D173, N174

Table 6: Residues Identified by Atomic Distance Analysis of 1C11-mCX3CR1

V_H Region	V_H Residues	V_L Region	V_L Residues
CDR-H1	N31,Y32	CDR-L1	Y31, S32, G33, N34, K36
CDR-H2	K54, S55, K57, F58, A60, N61, Y62, K67, G68	CDR-L3	Y98,Y100
CDR-H3	D101, P105, F106, Y108		
Non-CDR residues	S17, S30, A71, D76, S86		

Table 7: 1C11 Variable Region Residues Identified by Both Analyses of 1C11-mCX3CR1

mCX3CR1 Region	mCX3CR1 contacts w/V_H	mCX3CR1 Region	mCX3CR1 contacts w/V_L
N-terminus Domain	L10, F13, Y15, A19, E20, A21, Y23	N-terminus Domain	V28,T32
ECL2	K172, D173, D179, Y180, E182, Q185, E186	ECL1	L92, I93, S94, H95, E96
ECL3	K258, F259, K269	ECL2	D173,N174

Table 8: mCX3CR1 Residues Identified by Both Analyses of 1C11-mCX3CR1

mCX3CR1 Region	Residues
N-terminus	F13, Y15, A19, E20, A21, Y23, V28
ECL2	K172, D173
ECL3	K269

Table 9: Residues in mCX3CR1 that Interact with Both mCX3CL1 and the 1C11 mAb

SUPPLEMENTAL METHODS

Prediction of Protein-Protein Interactions

The predictions of the binding interactions between proteins were performed by individually inputting the sets of amino acid sequences into the alphafold predict tool in the ChimeraX program[2]. This tool was used to run the AlphaFold2-Multimer[3] implementation in ColabFold[4], an open-source software hosted on Google Colab. The models with the highest confidence, as determined by the AlphaFold2-Multimer program[2], were visualized in the ChimeraX program. The models were then assessed using the pTM and ipTM scores, which are error metrics generated by the AlphaFold2-Multimer program. pTM is a measure of the prediction accuracy of each individual protein chain, whereas ipTM is a measure of the prediction of the complex as a whole[8].

Obtainment of Amino Acid Sequences for AlphaFold2-Multimer Analysis

The human CX3CR1 and CX3CL1 amino acid sequences inputted into the alphafold predict tool were obtained from RCSB PDB Accession Code 7XBW[1]. In the PDB file, the human CX3CR1 and CX3CL1 were joined by a 28 residue linker (14 × Gly-Ser) and contained a Twin-Strep-Tag, a PreScission protease site, and a Flag Tag. The linker and tags were removed, and the sequences were aligned with the amino acid sequences of UniProtKB[5] Accession Number P49238 and UniProtKB[5] Accession Number P78423 to confirm the identities of human CX3CR1 and CX3CL1, respectively.

The mouse CX3CR1 amino acid sequence was obtained from UniProtKB[5] Accession Number Q9Z0D9 and was aligned with the inputted human CX3CR1 amino acid sequence. Using the alignment, the mouse CX3CR1 sequence was then shortened to match the length of the inputted human CX3CR1 amino acid sequence. The mouse CX3CL1 amino acid sequence was obtained from Novus Biologicals Catalog #NBP2-35038 Product Datasheet[6].

The V_H and V_L sequences of the anti-mCX3CR1 mAb (clone: 455.1C11) inputted into the alphafold predict tool were obtained from mRNA sequencing of the hybridoma by Absolute Antibody.

All of the amino acid sequences used are displayed in Table 1.

Contact Residue Analysis of mCX3CL1-mCX3CR1

To predict contact residues, two analyses were conducted on the pdb file of the highest confidence model in the ChimeraX program using the select contacts command. The select contacts command has two options for conducting contact analysis, and both were used to identify key residues of interest. The first option highlights residues with buried solvent-accessible surface area greater than or equal to 15.0 Å, the default lower bound. The second option highlights residues on mCX3CR1 and mCX3CL1 with atomic distance less than or equal to 3.5 Å, the default upper bound, from mCX3CL1 or mCX3CR1, respectively. For each method, 2 analyses were performed; residues were highlighted on mCX3CL1 that were in contact with mCX3CR1, and similarly on mCX3CR1 with mCX3CL1. After each analysis, the Show Sequence Viewer tool in the ChimeraX program was used to view the highlighted residues. The one-letter code and residue number were then manually recorded. After both analyses were conducted, the lists of contact residues for each individual chain from both analyses were analyzed to determine contact residues present in both lists.

Contact Residue Analysis of 1C11 mAb-mCX3CR1

To predict contact residues, two analyses were conducted on the pdb file of the highest confidence model in the ChimeraX program using the select contacts command. The select contacts command has two options for conducting contact analysis, and both were used to identify key residues of interest. The first option highlights residues with buried solvent-

accessible surface area greater than or equal to 15.0 Å, the default lower bound. The second option highlights residues on mCX3CR1 and the 1C11 mAb V regions with atomic distance less than or equal to 3.5 Å, the default upper bound, from the 1C11 mAb V or mCX3CR1, respectively. For each method, 4 analyses were performed; residues were highlighted on the 1C11 mAb V_H region that were in contact with mCX3CR1, and similarly on the 1C11 mAb V_L region with mCX3CR1, on mCX3CR1 with the 1C11 mAb V_H region, and on mCX3CR1 with the 1C11 mAb V_L region. After each analysis, the Show Sequence Viewer tool in the ChimeraX program was used to view the highlighted residues. The one-letter code and number were then manually recorded. After both analyses were conducted, the list of contact residues for each individual chain from both analyses were analyzed to determine contact residues present in both lists.

**Name**	**Sequence**
hCX3CR1	DQFPESVTENFEYDDLAEACYIGDIVVFGTVFLSIFYSVI<br>FAIGLVGNLLVVFALTNSKKPKSVTDIYLLNLALSDLLF<br>VATLPFWTHYLINEKGLHNAMCKFTTAFFFIGFFGSIFF<br>LTVISIDRYLAIVLAANSMNNRTVQHGVTISLGVWAAA<br>ILVAAPQFMFTKQKENECCGDYPEVLQEIWPVLRNVET<br>NFLGFLLPLLIMSYCYFRIIQTLFSSKNHKKAKAIKLILL<br>VVIVFFLFWTPYNVVIFLETLKLYDFFPSCDMRKDLRL<br>ALSVTETVAFSHCCLNPLIYAFAGEKFRRYLYHLYGKC<br>LAVL
hCX3CL1	QHHGVTKCNITCSKMTSKIPVALLIHYQQNQASCCKRA<br>IILETRQHRLFCADPKEQWVKDAMQHLDRQAAALTRN
mCX3CR1	STSFPELDLENFEYDDSAEACYLGDIVAFGTIFLSVFYA<br>LVFTFGLVGNLLVVLALTNSRKPKSITDIYLLNLALSDL<br>LFVATLPFWTHYLISHEGLHNAMCKLTTAFFFIGFFGGI<br>FFITVISIDRYLAIVLAANSMNNRTVQHGVTISLGVWAA<br>AILVASPQFMFTKRKDNECLGDYPEVLQEMWPVLRNS<br>EVNILGFALPLLIMSFCYFRIIQTLFSCKNRKKARAVRLI<br>LLVVFAFFLFWTPYNIMIFLETLKFYNFFPSCDMKRDLR<br>LALSVTETVAFSHCCLNPFIYAFAGEKFRRYLGHLYRK<br>CLAVL
mCX3CL1	QHLGMTKCEIMCGKMTSRIPVALLIRYQLNQESCGKRA<br>IVLETTQHRRFCADPKEKWVQDAMKHLDHQAAALTK<br>NG
V_H of anti-mCX3CR1 mAb (Clone: 455.1.1C11.23.16.20)	QVQLVETGGGLVRPGNSLKLSCVTSGFTFSNYRMHWL<br>RQFPGKRLEWIAVIAFKSDKFGANYAESVKGRFAISRD<br>DSKNSVYLEMSRLREEDTATYFCSRDGNGPFDYWGQG<br>TTLTVSS

V_L of anti-mCX3CR1 mAb (Clone: 455.1.1C11.23.16.20)	DIVMSQSPSSLTVSVGEKVTMSCKSSQSLLYSGNQKSY LAWYQQKPGQSPRLLIYWASTRESGVPDRFTGSGSGTD FTLTISGVKAEDLAVYYCQRYYGYPRTFGGGTKLEIK

Table 1: Sequences Used in Predictive Modeling

REFERENCES

1. Lu, M., Zhao, W., Han, S., Lin, X., Xu, T., Tan, Q., Wang, M., Yi, C., Chu, X., Yang, W., Zhu, Y., Wu, B., & Zhao, Q. (2022). Activation of the human chemokine receptor CX3CR1 regulated by cholesterol. *Sci. Adv.*, 8, eabn8048. https://doi.org/10.1126/sciadv.abn8048
2. Pettersen, E. F., Goddard, T. D., Huang, C. C., Meng, E. C., Couch, G. S., Croll, T. I., Morris, J. H., & Ferrin, T. E. (2021). UCSF ChimeraX: Structure visualization for researchers, educators, and developers. *Protein Sci.*, 30(1), 70-82. https://doi.org/10.1002/pro.3943
3. Evans, R., O'Neill, M., Pritzel, A., Antropova, N., Senior, A., Green, T., Zídek, A., Bates, R., Blackwell, S., Yim, J., Ronneberger, O., Bodenstein, S., Zielinski, M., Bridgland, A., Potapenko, A., Cowie, A., Tunyasuvunakool, K., Jain, R., Clancy, E., Kohli, P., Jumper, J. M., & Hassabis, D. (2021). Protein complex prediction with AlphaFold-Multimer. *bioRxiv*. https://doi.org/10.1101/2021.10.04.463034
4. Mirdita, M., Schütze, K., Moriwaki, Y., Heo, L., Ovchinnikov, S., & Steinegger, M. (2022). ColabFold: making protein folding accessible to all. *Nat Methods*, 19(6), 679-682. https://doi.org/10.1038/s41592-022-01488-1
5. The UniProt Consortium. (2023). UniProt: the Universal Protein Knowledgebase in 2023. *Nucleic Acids Research*, 51(D1), D523–D531. https://doi.org/10.1093/nar/gkac1052
6. Novus Biologicals. (2022, September 26). Product Datasheet Recombinant Mouse CX3CL1/Fractalkine Protein NBP2-35038-5ug.
7. Madeira, F., Pearce, M., Tivey, A. R. N., et al. (2022). Search and sequence analysis tools services from EMBL-EBI in 2022. *Nucleic Acids Research*, 50(W1), W276-W279. https://doi.org/10.1093/nar/gkac240
8. O'Reilly, F. J., Graziadei, A., Forbrig, C., Bremenkamp, R., Charles, K., Lenz, S., Elfmann, C., Fischer, L., Stülke, J., & Rappsilber, J. (2023). Protein complexes in cells by AI-assisted structural proteomics. *Molecular systems biology*, 19(4), e11544. https://doi.org/10.15252/msb.202311544
